# The role of gene to gene interaction in the breast’s genomic signature of pregnancy

**DOI:** 10.1038/s41598-021-81704-8

**Published:** 2021-01-29

**Authors:** Pedro J. Gutiérrez-Díez, Javier Gomez-Pilar, Roberto Hornero, Julia Martínez-Rodríguez, Miguel A. López-Marcos, Jose Russo

**Affiliations:** 1 IMUVA Mathematical Institute, University of Valladolid, Valladolid, Spain; 2 Faculty of Economics, University of Valladolid, Valladolid, Spain; 3 Biomedical Engineering Group, University of Valladolid, Paseo de Belén, 15, 47011 Valladolid, Spain; 4 Centro de Investigación Biomédica en Red en Bioingeniería, Biomateriales Y Nanomedicina (CIBER-BBN), Valladolid, Spain; 5 Faculty of Science, University of Valladolid, Valladolid, Spain; 6 The Irma H. Russo, MD Breast Cancer Research Laboratory, Fox Chase Cancer Center - Temple University Health System, Philadelphia, USA

**Keywords:** Cancer genomics, Cancer genetics, Gene regulation, Genome informatics

## Abstract

Full-term pregnancy at an early age confers long-term protection against breast cancer. Published data shows a specific transcriptomic profile controlling chromatin remodeling that could play a relevant role in the pregnancy-induced protection. This process of chromatin remodeling, induced by the breast differentiation caused by the first full-term pregnancy, has mainly been measured by the expression level of genes individually considered. However, genes equally expressed during the process of chromatin remodeling may behave differently in their interaction with other genes. These changes at the gene cluster level could constitute an additional dimension of chromatin remodeling and therefore of the pregnancy-induced protection. In this research, we apply Information and Graph Theories, Differential Co-expression Network Analysis, and Multiple Regression Analysis, specially designed to examine structural and informational aspects of data sets, to analyze this question. Our findings demonstrate that, independently of the changes in the gene expression at the individual level, there are significant changes in gene–gene interactions and gene cluster behaviors. These changes indicate that the parous breast, through the process of early full-term pregnancy, generates more modules in the networks, with higher density, and a genomic structure performing additional and more complex functions than those found in the nulliparous breast.

## Introduction

Breast cancer, the most common cancer in women both in the developed and less developed world, is a long-known disease. As documented in the Edwin Smith papyrus, evidence on the occurrence of breast cancer dates back to 3000–1500 years BC^1^, and, since then, this disease has been the subject of medical study. A common denominator for the risk of developing breast cancer, a complex disease due to the uncontrolled growth of cells unique and specific to the breast, is the reproductive history. As early as the 1700s, it was observed that increased breast cancer incidence and mortality were associated with nulliparity, a fact reported by Bernardino Ramazzini, who attributed the phenomenon to the childlessness of nuns in Italian convents^2^. Since then, early full-term pregnancy has been associated with a significant decrease in the risk of breast cancer in postmenopausal women, as reported by several epidemiological studies^3–5^. On the other hand, late pregnancy and nulliparity have been associated with increased risk^6^.

Different hypotheses have emerged to explain the mechanisms underlying this relationship, among which the mediation of protection induced by environmental changes^7^ and immunological alterations stands out^8^. Previous studies have revealed important morphological, functional, genomic and transcriptomic changes associated with breast differentiation^9–11^, which is influenced by the milieu hormonal complex produced by the placenta and fetus^12^. These changes, ultimately, regulate and induce a specific profile that confer permanent protection against the risk of cancer^13,14^. In this respect, we have provided evidence supporting that early pregnancy changes the genomic signature causing a differentiation of the lobular structures of the parous breast from those of the nulliparous one^13,15,16^. Over the last few years, we have also clearly shown that the first full-term pregnancy (FFTP) induces a specific genomic signature in the human breast in postmenopausal and premenopausal women that explains this protective effect^17–26^. Taking the detection for postmenopausal women of a specific genomic signature induced by early FFTP^17,18,27^ as the starting point, several enriched biological pathways and processes were identified. In the case of down-regulated genes, enriched biological processes included IGF-like growth factor signaling, somatic stem cell maintenance, and apoptosis. Concerning pathways enriched by down-regulated genes, the most significant ones represent proteins that are highly expressed in malignancies, such as the insulin, Wnt, and integrins signaling pathways, MAPK, cytokine-cytokine receptor interaction, tight junction, and focal adhesion. Processes enriched by up-regulated genes comprised cell-substrate junction assembly, RNA related processes, and differentiation and development of epidermis and ectoderm. On the other hand, those pathways made up of genes upregulated are involved in breast cancer estrogen signaling, cell communication, and mRNA processing machinery. Finally, we have shown that these changes are tightly associated with the remodeling of chromatin associated to FFTP^18^. The evidence of changes in chromatin structure caused by FFTP, which constitutes the starting point of our research and guides the interpretation of the results, comes from multiple and complementary perspectives. The widest, applicable to the regulation of eukaryotic gene expression and not only to FFTP, confirms that chromatin remodeling, methylation patterning, and transcriptional regulation are closely connected and can be considered as a unique complex mechanism. In this respect, recent research has also shown that chromatin structure changes precede and dictate patterns of DNA methylation and regulate gene transcription^28,29^, through mechanisms and processes also documented for FFTP by Russo and colleagues^18,24,25^. More specifically, in^18^, the authors carried out transcriptomic analyses revealing the presence, in core needle biopsies of the parous breast, of 267 upregulated probesets that comprised genes controlling chromatin organization, transcription regulation, splicing machinery, mRNA processing, and 48 long non coding RNA (lncRNAs). As this research team confirmed in the study of Santucci-Pereira and colleagues^24^, the identified transcriptomic changes induced by early FFTP were closely associated with the activation of genes controlling chromatin remodeling, and the expression of Histone 3 at lysine 9 and 27, with the morphological manifestation of an increase in heterochromatin formation that remained in the menopausal breast. This finding is in concordance with the pivotal role played by chromatin remodeling in determining methylation and gene transcription, identified by ^28^ and ^29^and also shown in^25^ for FFTP. This research finds that lncRNAs are activated during the early FFTP, an additional proof that epigenetic changes are taking place through chromatin remodeling. In general, it is known that lncRNAs are responsible for chromatin remodeling ^30–38^: lncRNAs bind to transcripts in the nucleus as they emerge from the replication fork of the DNA, and recruit enzyme complexes to induce epigenetic changes at chromatin remodeling loci. The associated chromatin remodeling proteins then modify the local chromatin and DNA, suppressing gene expression. One such modification is methylation of the DNA, which presumably occurs when the lncRNAs direct enzymes -such as the DNA methyltransferase DNMT3a- to targeted spots on the genome. Alternatively, lncRNAs can direct modifications of nearby histones, usually in the form of methylation of the histone tail, and have a crucial role in regulating gene transcription and post-transcriptional regulation through chromatin remodeling ^30–38^.

The abovementioned changes affecting gene clusters and pathways, caused by parity-induced chromatin remodeling, have been determined through the previous identification of the up/down regulation of genes. For this set of up/down regulated genes, individually identified, if some relationship between a specific group of genes arose (according to biomedical and/or statistical criteria such as Gene Ontology and Gene Set Enrichment Analysis), the existence of a cluster or module within the global genetic network was deduced. However, here we show that modifications in the joint behavior of clusters of genes coexist with, and may be independent of, changes in the expression of genes individually considered, constituting an additional and important characteristic of the genomic signature associated to parity. This is consistent with the existence of a genomic signature centered on chromatin remodeling^18^. Given that parity alters the expression of genes regulating chromatin remodeling^19–24^ and, at the same time, chromatin remodeling determines the genomic expression, the existence of complex changes involving sets of genes is highly likely^24,39^. The present work presents a novel approach to identify these changes, focusing on pure joint behaviors by applying concepts and techniques taken from Information (IT) and Graph (GT) Theories, Differential Co-expression Network Analysis (DCNA), and Multiple Regression Analysis (MRA). This study complements gene-by-gene individual approaches with gene-to-gene cluster approaches: we directly inspect the existence of changes, otherwise ignored, in the behavior of gene clusters associated to FFTP, proving their presence, and identifying networks specific for nulliparous and for parous women, or changing from nulliparous to parous.

## Results and discussion

Using the database published in the literature^27^, deposited in the Gene Expression Omnibus database (GSE26457), we analyze the expression level for 18,653 probes in 42 nulliparous and 71 parous women.

### Gene-by-gene analysis

Through the gene-by-gene analysis of expression level data under different complementary criteria, we obtain new information characterizing the genomic signature of parity. In particular, to show that the genomic signature of pregnancy is characterized by changes at the individual gene level which coexist with, and are independent of, changes at the gene cluster level, we compute three different magnitudes for each probe. These magnitudes summarize different aspects of each probe data series, and are the following: the mean value of the expression level (ME); the area under the cumulative distribution function (CD); and the Shannon entropy (SE).The use of ME allows upregulated and downregulated genes to be identified in parous with respect to nulliparous. This criterion provides information on the (average) activity of each gene in each group, and then on the changes in each gene activity between nulliparous and parous. This is not the only possible change. Indeed, in a first statistical inspection of the expression level data, we found a high number of probes with different statistical distributions between nulliparous and parous, but showing no change in the mean expression value. This suggests that there exist genes whose average activity remains constant across groups, but which attain this constant activity through different channels and stimuli in nulliparous and parous. In addition, we also identified significant modifications in the interrelationships between probes that are not associated with changes in their mean expression values, pointing to the existence of a rearrangement of gene functions.

Regarding these questions and to properly situate the contribution and scope of our three gene-by-gene criteria, especially those of CD and SE, we first explain how the dataset was obtained, pre-processed and previously analyzed. In this respect, all this information is exhaustively detailed in^27^ and ^17^, where the same dataset is used to study the role of a selected and reduced number of genes. As explained in these two papers, the used data meet the strongest reliability requirements, and thus allow the effects associated to FFTP to be correctly isolated and identified. More specifically, these methods include strict protocols in data collection and cRNA preparation, the correction for background noise (implementation of Affymetrix PLM analysis), the removal of possible batch-effects (ComBat), and normalization. Once the accuracy, robustness and reliability of the dataset to inform on changes caused by FFTP is ensured^40,41^, the interpretation of the proposed novel CD and SE criteria (in general of our gene-by-gene analyses) can now be clarified.

Concerning CD, our interpretation is based on a well-established fact in genomics, namely the control of gene expression levels through complex gene networks and pathways^42–47^. From a mathematical perspective, the presence of such gene clusters or networks determining gene expression levels can be envisaged as stable functional relationships between the genes involved^48–52^. Accordingly, for each gene, its expression levels are a direct consequence of the (hidden) relationships with the expression levels of other genes, which must be interpreted as causes, channels or stimuli determining the expression levels of the considered gene. In terms of the CD criterion, for each probe, the cumulative distribution function obtained from the data series of its expression level is necessarily the result of these causes, channels or stimuli. Therefore, once noise, batch effects and other confounding informative elements have been removed, modifications in the cumulative distribution function of the probe between nulliparous and parous only appear when these channels, causes or stimuli determining its expression levels (i.e., the way this gene is related to others) change across groups. The underlying assumption is that these changes lead to distinct statistical distributions of the probe in the two groups, and then to different areas under the cumulative distribution function.

CD allows us to identify these genes with no changes in their mean expression levels, but whose statistical distribution has changed from nulliparous to parous, probably responding to distinct biological causes and stimuli, and thus related differently to other genes. Associated to this aspect, SE provides the amount of information inherent to the data series of each probe and group. For each probe, it is assumed that its expression level is a random variable with a finite set of possible outcomes. It therefore makes sense to compute the Shannon entropy of the series. This Shannon entropy measures, for each probe, the "amount of information" contained in the data series of its expression levels, and can be interpreted as a direct consequence of the number and complexity of functions associated to the gene.

**Figure 1:**
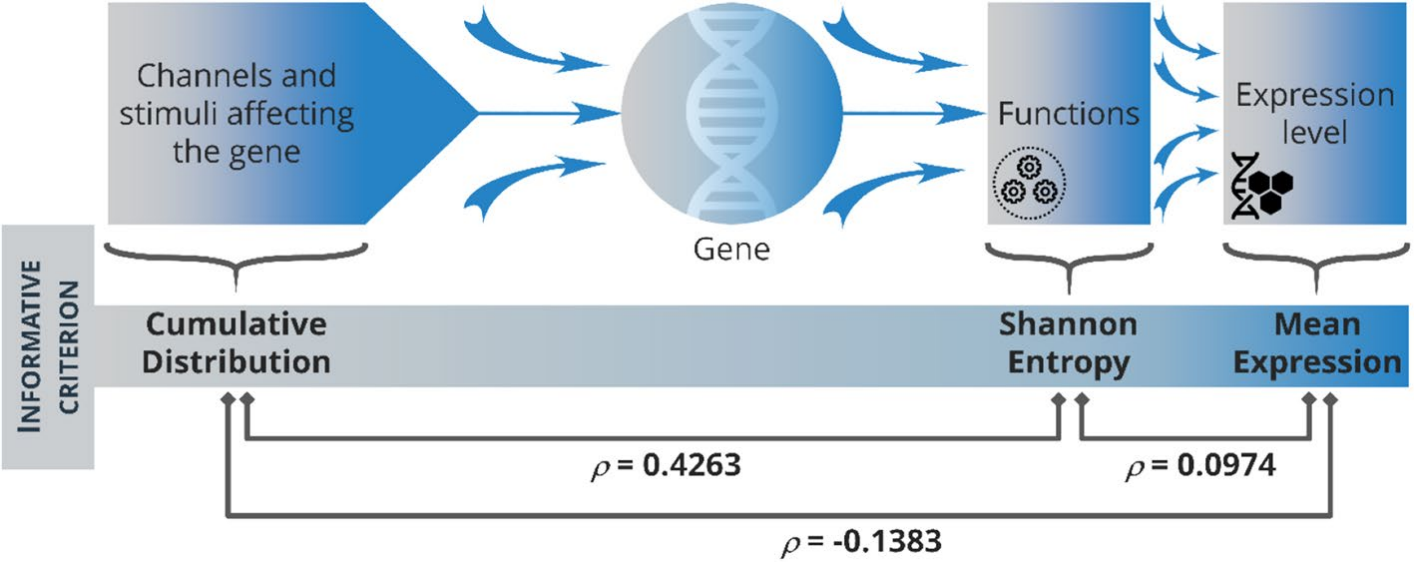
Diagram summarizing the meaning of the Cumulative Distribution, Shannon Entropy and Mean Expression level criteria, as well as the relationship between them.

As happened for the CD criterion, our interpretation of the SE criterion is also based on a well-established genomic fact, in this case the univocal relationship between the gene expression levels and the biological functions of the considered cell at the proper time^52–54^. Then, if the expression levels of a gene are the means through which the gene develops its functions, from the analysis of the series of the gene level expressions, it is possible to investigate some aspects of the gene functions and to ascertain whether or not these functions change between nulliparous and parous. As a novel criterion, we propose to use the SE for this purpose, since it measures (in bits) the amount of information provided by a data series when it works as an encoded message. This is our case: although the SE can measure information, noise or uncertainty depending on the underlying phenomenon that originates the data series, the non-existence of noise and other misleading informative elements in our dataset ensures that the series of expression levels is the means through which the gene carries out its functions^55^. Its SE therefore provides the information transmitted by the gene, and thus, in biomedical terms, we can interpret that when the gene performs additional and/or more complex functions, we find a higher entropy. Each gene-by-gene perspective provides information on a different dimension of the existing changes between nulliparous and parous. These changes can appear simultaneously or, on the contrary, in isolation, thus providing complementary information. This complementarity is inversely related to the absolute value of the correlation between the considered gene-by-gene measures (see Fig. 1 for a diagram illustrating the meaning of CD, SE and ME criteria, as well as the relationship between them).

**Figure 2:**
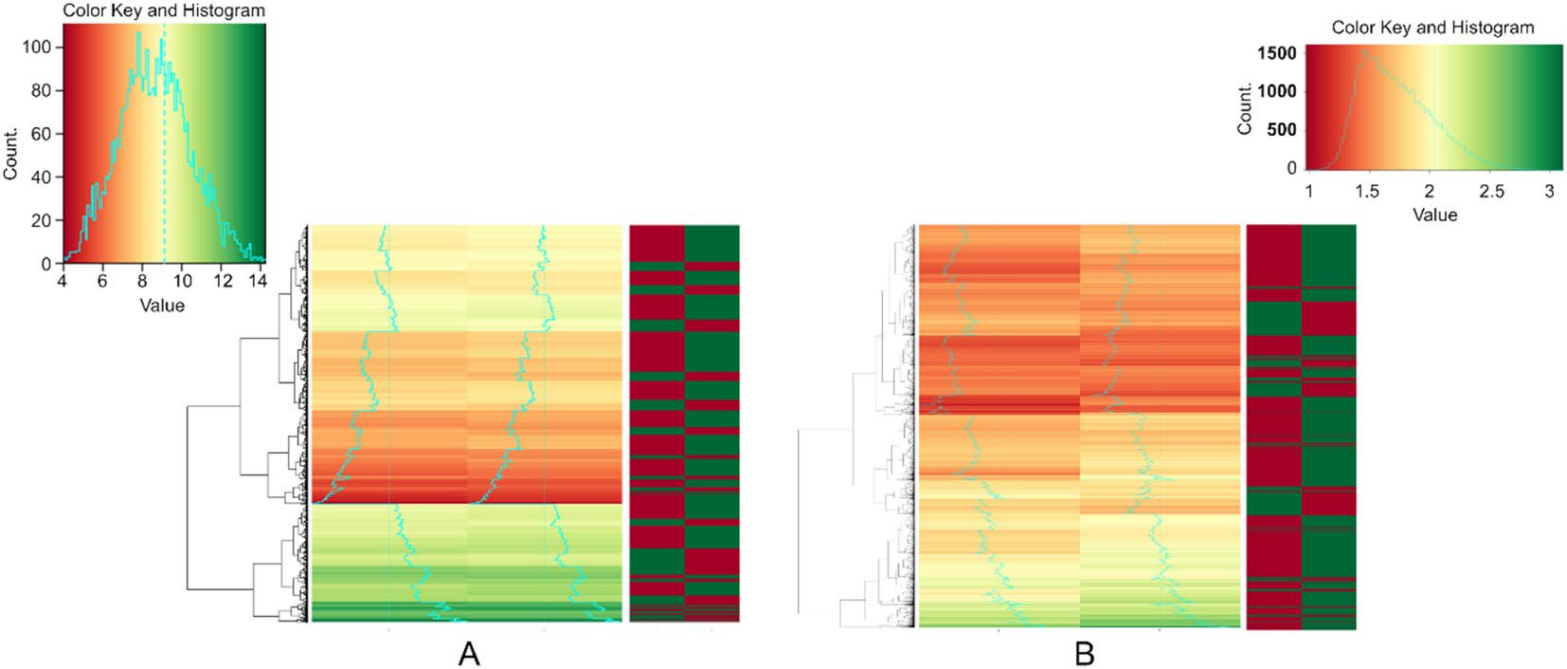
Genetic signature of pregnancy through gene-by-gene analysis. Heatmaps. **(A)** Heatmap of all 2123 probes found differentially expressed (adjusted *p *value < 0.05) between nulliparous (left column) and parous (right column). Two heatmaps are presented to show specific features of the FFTP genomic signature associated with up/down regulation. Each horizontal line corresponds to the same probe in both heatmaps. The left heatmap clusterizes according to similarity of expression levels (red to green: low to high expression level). The right heatmap shows downregulation (red) and upregulation (green). As the histograms (blue curves) depict, upregulation for parous is more frequent in the central orange/yellow region (low/intermediate expression values in nulliparous). For high expression values (bottom region), downregulation for parous is slightly more frequent than overregulation. There is a general tendency to upregulation in parous, also found by ^27^, with an up/down regulation ratio of 2.17. **(B)** Heatmap of changes in entropy for the 18,022 probes showing significant changes (*p *value < 0.05). Cluster analysis shows a generalized gain in entropy induced by FFTP, which is not related to the entropy level in nulliparous (histograms/blue curves): The redefinition of the channels and stimuli reaching genes and their performance of additional and more complex functions as a consequence of FFTP homogenously affects all genes.

Our findings after applying these three gene-by-gene analyses confirm, from different and complementary dimensions, the existence of a genomic signature associated to pregnancy and the central role played by chromatin remodeling. Concerning the identification of differentially expressed genes, several tests and criteria have been applied in the literature, depending on the research characteristics and goals. Jointly with the *p *values arising from a wide variety of tests, additional criteria are usually imposed to further select differentially expressed genes and reduce their number. The most frequent is to include a threshold value for the log2 fold change. Since the detailed biological analysis of gene functions and pathways is only possible for a reduced number of genes, the fold change cut-off is applied to remove genes which achieve statistical significance but that, according to the biological and medical criteria of the researchers, do not reach a sufficient difference in an absolute sense as to justify the costly analysis of their functions. On this point, it is worth noting that, with the same data set, specific clustering, Gene Ontology, and Gene Set Enrichment analyses have been implemented in^27^ on a selected reduced set of genes of interest after imposing restrictive thresholds for the *p *value and the log2 fold-change. In our case, the objective of the research is to show how the whole genetic profile changes as a consequence of the chromatin remodeling associated to FFTP, the identification and study of specific genes having a relatively minor interest. For this reason, we have considered the adjusted *p *value as the unique criterion to conclude the presence of up/down regulation. More specifically, after running a two-sample t-test with non-equal variances, we implemented a False Discovery Rate (FDR) control to correct the multiple testing problem by applying the BH procedure, and calculated the adjusted *p *values. Regarding gene activity level in parous, 669 probes are downregulated and 1454 upregulated with respect to nulliparous [adjusted *p *value < 0.05]. As explained previously, our main objective concerning ME is to show the existence of global changes in the map of gene expression, analyzing the same dataset in^27^ and^17^ from different but complementary perspectives. In these papers, the authors applied restrictive criteria to identify a reduced number of differentially expressed genes of particular interest for their research. With this set, they generated a clustered heatmap by using uncentered Pearson correlation as a similarity measure, and average linkage as the agglomeration method, obtaining significant evidence on the existence after FFTP of changes in genes involved in chromatin remodeling. Here, to gain new evidence on the existence of generalized changes in the map of expression levels associated to FFTP and chromatin remodeling, and to characterize them, we implement an alternative perspective, using the Euclidean measure to obtain the distance matrix, and the complete agglomeration method for clustering. In accordance with our objective, we applied our analysis to all probes showing statistically significant changes in their expression levels (adjusted *p *value < 0.05), and not only to those selected by restricting biomedical criteria as in^27^ and^17^. Through our alternative clustering procedure, we provide new evidence on the presence of a change in the global map of expression levels, summarized in Fig. 2. In the upper row, on the left, the clustered heatmaps of all 2123 differentially expressed probes between nulliparous (left column) and parous (right column) are depicted. The left heatmap clusterizes according to the similarity of expression levels (red to green: low to high expression level), and the right heatmap shows downregulation (red) and upregulation (green) in parous with respect to nulliparous. Each horizontal line corresponds to the same probe in both heatmaps. These two heatmaps provide information on the relationships between expression values in nulliparous and up/down regulation in parous, i.e., on the structure of the changes in expression levels caused by FFTP. More specifically, we observe that upregulation for parous is more frequent for the nulliparous upper and central red/orange/yellow region, that is, when nulliparous present low/intermediate expression values. For high expression values in nulliparous (bottom region in green), downregulation for parous is slightly more frequent than overregulation. There is a general tendency to upregulation in parous, also found by Russo and colleagues^27^, with an up/down regulation ratio of 2.17. This structure underlying the changes in expression levels for the whole set of probes is also represented by the histograms, depicted as a blue line. In the color-key subfigure, the histogram of all the expression levels for both nulliparous and parous is depicted jointly with the color code. Additionally, it is possible to calculate the specific histogram or each group of women. After applying the clustering algorithm, this order in the color-key is altered, and the initial histogram-blue line is divided according to the obtained clusters. Therefore, by comparing between groups how the different sections of the clustered histogram are located with respect to the dashed central line, it is possible to extract conclusions about the structure underlying the observed changes in expression levels. In particular, the change in the position of the blue line across groups helps to relate up/down regulation in parous with a specific value of the expression level for nulliparous: When, for a range of colors in nulliparous, the blue line for parous is relatively more to the right/left, there appears up/down regulation. In addition, the higher the shift of the blue line/histogram section, the higher the change in expression level. Finally, it is also possible to assess to what extent changes in expression levels lead to a different structure/distribution. In our case, the histogram for parous has a lower dispersion: with respect to nulliparous, the blue line shifts to the right at the range of colors red and orange, and to the left at green ranges. This implies that, at the genome global level, FFTP entails a general tendency not only to up regulation, but also to a reduction in the range of expression levels. For those genes involved in chromatin organization, we identify the same modifications found by Russo and colleagues^19^ (Table S1 in the Supplementary Material). Coherently, if parity is accompanied by chromatin reorganization, it should imply significant changes in expression levels of other genes, and then in the way they are interrelated, the channels and stimuli affecting the genes, and the number and/or complexity of their functions. This is what we obtain by applying CD and SE and running the Kolmogorov–Smirnov and Wilcoxon tests to detect changes in the statistical distribution of each probe between groups. Given that the evidence on the reliability of the use of FDR control in the Kolmogorov–Smirnov and Wilcoxon tests is not clear^56–58^, we present both the analyses in terms of *p *values and adjusted *p *values. For *p *values < 0.05, CD identifies 4939 probes (26.5% of the total number) modifying their statistical distribution from nulliparous to parous. When the FDR control is implemented, for an adjusted *p *value < 0.05, we find 1986 probes (10.64%). This suggests that they are associated to genes responding to distinct channels and stimuli after FFTP, and then differently related with other genes. To analyze changes in SE, we implement bootstrapping. SE is a magnitude with no statistical nature for which direct statistical tests or interpretations are not possible, and the implementation of bootstrapping allows the statistical significance of changes in SE to be modeled. For each probe and group, 1,000 resamples were randomly selected, and their SE calculated. After computing the mean for each probe in the different groups, a t-test was run to conclude statistically significant differences (adjusted *p *value). From nulliparous to parous, 13,160 probes (70.6% of the total number) gain entropy, suggesting that associated genes perform additional and/or more complex functions, while 4727 (25.3%) present entropy descents. As for ME, the two heatmaps on the right in Fig. 2 provide information on the structure underlying these changes in SE. In particular, from the analysis of the histograms depicted by the blue curves, we can conclude that there exists a general tendency to gain entropy after FFTP, and that this tendency is not related to the entropy level in nulliparous. According to our interpretation, the redefinition of the channels and stimuli reaching genes and their performance of additional and more complex functions as a consequence of FFTP homogenously affects all genes.

**Figure 3:**
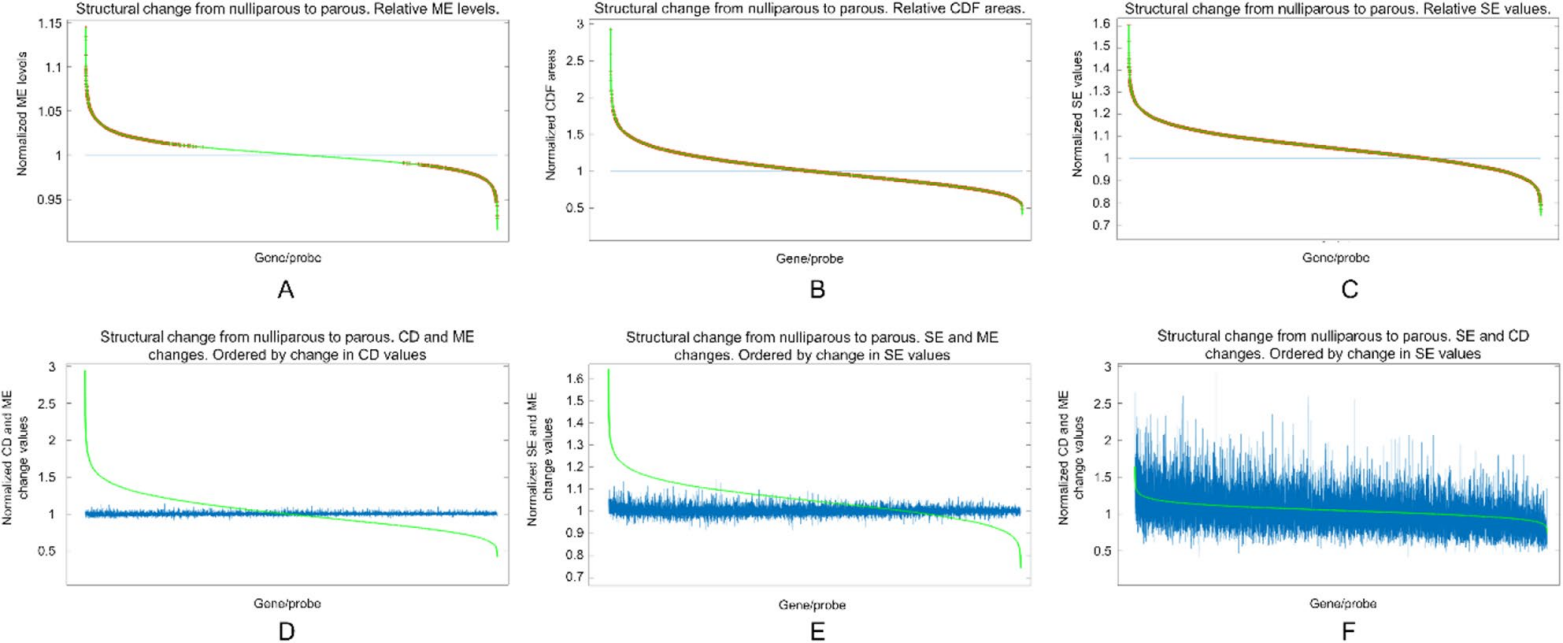
Structural changes associated to FFTP, gene-by-gene analyses. Upper row: The X axis represents each of the 18,653 probes, and the horizontal line at value 1 represents, for each probe in the X axis, its normalized expression value for nulliparous. To show the existence of a specific genomic signature for parous and its different dimensions, the ordered relative mean expression level for each gene in the parous group is depicted by the green curve. The higher the changes associated to FFTP, the more distant the green curve. Statistical significance is marked in red. **(A)** Restructuration of mean expression levels and general tendency to upregulation after FFTP. **(B)** Existence of distinct channels and stimuli affecting the genes after FFTP. **(C)** Changes in the number and complexity of functions performed by each gene after FFTP. **Bottom row:** The X axis represents each of the 18,653 probes. For each pair of criteria, the simultaneous changes are depicted. (**D**) Simultaneous changes in CD and ME (ρ = -0.1383). (**E**) Simultaneous changes in SE and ME (ρ = 0.0974). (**F**) Simultaneous changes in SE and CD (ρ = 0.42633).

These 3 gene-by-gene criteria allow the structural global changes in the map of gene expression to be characterized from different and complementary perspectives. By taking the nulliparous values as the reference, we can obtain a simple but very informative graphical representation of these structural changes. More specifically, for each criterion separately, let the X axis represent each of the 18,653 probes, and the horizontal line at value 1, the normalized expression value of each probe in the X axis for nulliparous. To show the existence of a specific genomic signature for parous and its different dimensions, the ordered relative value of ME, CD and SE for each gene in the parous group is depicted by the subsequent green curve. Obviously, the higher the changes associated to FFTP, the more distant the green curve from the horizontal line. As Fig. 3a shows, there exists a clear restructuration of mean expression levels, statistical distributions and Shannon entropies after FFTP, with a general tendency to upregulation and gains of entropy. We have also represented the statistical significance for each criterion with a red boundary.

The independence and coexistence of the three changes identified by our three gene-by-gene criteria are quantified by the correlation between the obtained measures. ME and SE measures are almost totally independent, with a very low correlation (ρ = 0.0974, Pearson correlation coefficient), so changes in gene activity level seem to have no relation to alterations in number and type of gene functions. Values obtained by applying ME and CD also have a low correlation (ρ = -0.1383), suggesting that changes in gene activity associated to parity do not seem to be related to changes in channels and stimuli reaching the gene. Indeed, for a *p *value < 0.05, among the 4939 probes changing their statistical distribution, there are 2903 probes (58.78%, roughly half of the total number) whose mean expression levels do not suffer significant alterations between groups. When FDR control is implemented and an adjusted *p *value < 0.05 is considered, we obtain 225 probes (11.3%). Finally, as expected, the correlation between the changes in SE and CD is considerably higher (ρ = 0.4263), pointing to simultaneity between the performance of additional and/or more complex tasks and the alteration of channels and stimuli affecting the gene. These correlations are visualized in Fig. 3b, where the relative changes of the distinct pairs of criteria are simultaneously depicted to visualize the correlation degree.

To correctly assess these results arising from the analysis of the ME, CD and SE criteria, it is necessary to take into account the scope and limitations of the used data, criteria, and measurements. In this respect, although the microarray technology constitutes a substantial improvement on the former traditional methods to study genomic profiles^59,60^, the reliability and validity of the microarray data on expression levels is subject to limitations. Given the complexity of the inspected biological phenomena, the conceptual and technical restrictions of microarray technology, and the unknowns concerning the implemented statistical sampling method, the evidence arising from the microarray data and its interpretation must be envisaged as likely and plausible but not definitive^61–63^. In addition to these troublesome technical and statistical issues, there exists biomedical evidence showing that, when interpreting microarray data on expression levels, there can exist unidentified confounding or shadow factors as well as unrecognized indirect relationships between expression levels (genes that mutually affect each other, not directly but through a third biological variable). The total removal of these problematic features is not possible, but their consequences on the research goal can be minimized by making use of complementary evidence coming from other perspectives. In this respect, to obtain additional confirmation and validation of our interpretations of the analyses carried out for the ME, CD and SE criteria, we incorporate results from gene ontology (GO) analysis. This allows the existence of confounding/shadow factors and indirect relationships to be inspected, clarifying the relative importance of the chromatin remodeling caused by FFTP. Concerning the ME criterion, the relevant GO results for this dataset are those described by Russo and colleagues^18,27^, who carried out a GO analysis for the found differentially expressed genes. As we explained in the previous section, their findings on the role played by chromatin remodeling after FFTP constitute the starting point of our research. They find a significant list of biological processes and functions tightly associated to chromatin remodeling, summarized in the introduction section. For our purposes, it is worth noting that these processes and functions, which include but are not limited to chromatin remodeling, are not related to possible shadow/confounding factors (such as fertility-infertility) or to the existence of hidden cross-effects. On the contrary, these researchers^18,27^ show that, although the number of differentially expressed genes related to chromatin remodeling is low compared with other genes (see Table S1), the morphological studies clearly confirm that chromatin remodeling took effect. In this respect, they identify cell expressing heterochromatin -a tightly packed form of DNA playing an important role in the expression of genes- and discuss in detail how the expression of the genes not directly related to chromatin remodeling that are differentially expressed in parous women, are the product of the chromatin remodeling process (for instance genes controlling cell differentiation). In addition, linked to development biology mechanisms, Russo and colleagues^18,27^ also find evidence of differentiation processes in the embryo due to the chromatin remodeling caused by FFTP, which also explains the simultaneous morphological irreversible changes in the breast as well as in the expression levels of the associated genes and pathways. This strong evidence on changes in chromatin remodeling caused by FFTP also appears when the GO analysis is applied to the other two metrics. For the CD and SE criteria, the GO analyses clarify and complement the results obtained for the differentially expressed genes. As Table S1 shows, there exist several genes involved in chromatin remodeling and whose CD and/or SE significantly change between nulliparous and parous. This table also reflects the complementarity of the three metrics used, especially with respect to the ME criterion: among the considered 27 genes related to chromatin remodeling, 9 (33%) significantly change between nulliparous and parous according to the CD or SE criteria, but not with respect to the ME metric. This suggests that the chromatin remodeling associated to FFTP can act in different dimensions, and not only through the modification of (mean) expression levels. The identification of the biological processes, molecular functions and cellular components linked to the genes changing CD and SE metrics, enabled by the GO analysis, allows us to inspect this hunch. In this respect, for those genes significantly modifying their CD, we identify a very high number of processes (over 50), functions (over 10) and components (over 30) related to cell differentiation, RNA metabolism, splicing, methylation, cell cycle, programmed cell death, and proliferation, all of them strongly related to chromatin remodeling. Concerning the possible existence of shadow or confounding factors and hidden cross-effects, GO analysis only identifies one molecular function, but no biological processes or cellular components. In addition, this unique function is related to G protein Couple receptor, and although may be an indication of receptors activated by hormones, is extremely general in cellular function to be attributed to shadow factors such as fertility or sterility. The GO analysis for the SE criteria provides similar results, being the main additional feature the identification of a higher number of biological processes related to the female pregnancy course (consistently with our interpretation of this criterion), and no signs of confounding factors such as infertility, or hidden cross-effects. Although the presence of shadow factors or unidentified indirect gene relationships in explaining the observed changes in the genomic map after FFTP can not be totally excluded, the results obtained from GO analysis under the considered 3 criteria (ME, CD and SE), strongly suggest the existence of a chromatin remodeling process playing a crucial and irreversible role.

In summary, our analyses of the gene-by-gene criteria support the idea that, after FFTP, the expression of genes controlling chromatin remodeling is modified (ME), and, as a consequence of chromatin reorganization, there also appear the logical induced modifications in the way genes express and interrelate (CD and SE), giving rise to additional dimensions characterizing the specific genomic signature of pregnancy (Figs. 2 and 3). Concerning the GO results arising from the ME criterion, we refer the interested reader to Russo and colleagues^18,27^, where an exhaustive analysis is carried out. For the two new applied criteria, CD and SE, the GO results are those specified in the Supplementary Material (Tables S7–S15).

### Gene-to-gene changes

Given the bidirectional relationship between chromatin and genomic expression, gene-to-gene analysis provides a complementary approach of the conventional gene-by-gene analysis to characterize the genomic signature of pregnancy, as well as the interrelations between the expression levels. More specifically, we inspect the same questions from a different perspective, focusing on the joint behavior of gene clusters and identifying changes in gene-to-gene interactions. We begin with the simplest case, studying correlations between probes by combining GT^64,65^ and DCNA^66^. We consider all the possible pairs between the 18,653 probes, computing their correlations for parous and nulliparous. These correlations are summarized in Table 1.

**Table 1:** Size of the networks for each threshold for parous and nulliparous groups.

Correlation range	Parous		Nulliparous	
	# of correlations	% of correlations	# of correlations	% of correlations
**(0.8, 1]**	**244,854**	**0.14**	**208,870**	**0.12**
(0.6, 0.8]	3,422,562	1.97	3,262,154	1.87
(0.4, 0.6]	12,553,221	7.22	12,735,638	7.32
(0.2, 0.4]	29,429,668	16.92	29,728,728	17.09
[-0.2, 0.2]	89,671,694	51.55	89,564,233	51.49
[-0.4 -0.2)	26,850,862	15.43	27,075,694	15.56
[-0.6, -0.4)	10,291,345	5.92	9,793,748	5.63
[-0.8, -0.6)	1,487,726	0.85	1,571,718	0.90
**[-1 -0.8)**	**5946**	**0.00**	**17,095**	**0.01**
Total	173,957,878	100	173,957,878	100

High-correlation networks are marked in bold.

**Figure 4:**
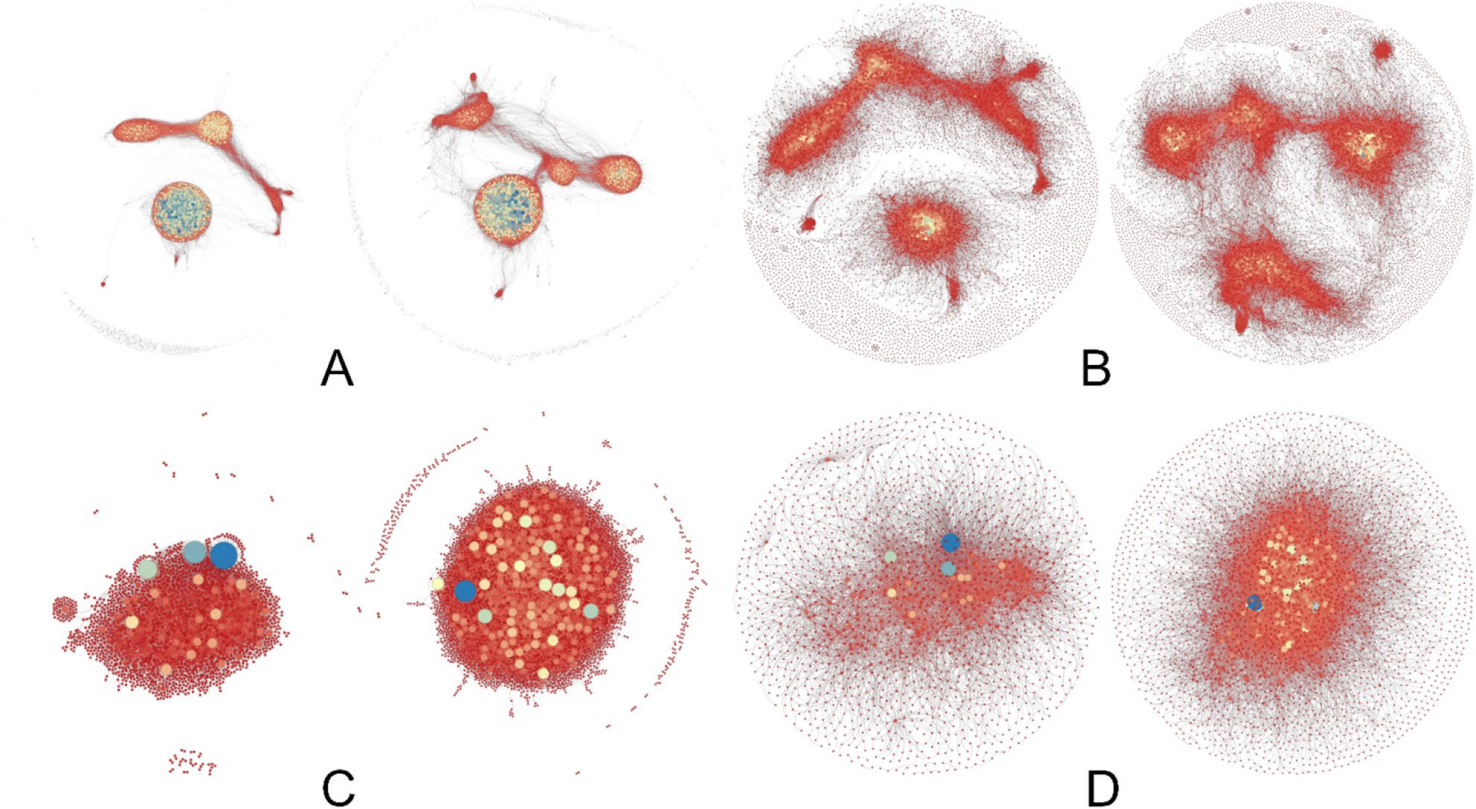
High correlation networks depicted by ForceAtlas2 (**a**,c) and Fruchterman-Reingold (**b**,**d**). For each figure, the network on the left shows correlations in parous, and the network on the right in nulliparous. Each probe is represented by a node, and when the correlation ρ between this probe and any other verifies abs(ρ) > 0.8, a link is depicted. In addition, the higher this value, the smaller the distance between the two linked probes. The size of each node/probe is proportional to the number of correlations verifying abs(ρ) > 0.8 that the probe presents with all the others. Color also varies (blue for nodes for higher number, yellow for intermediate, and red for lower). **(a, b)** High positive correlation networks, 1 < ρ ≤ 0.8, ForceAtlas2 (a), Frutcherman-Reingold (b). **(c, d)** High negative correlation networks, -1 < *ρ* ≤ -0.8, ForceAtlas2 (c), Frutcherman-Reingold (d). In all cases, the different network shapes and probe sizes and colors between groups are coherent with changes in the correlation structure after FFTP caused by variation in chromatin remodeling.

As clearly appears, the structure of the correlations is very different between groups (Fig. 4). Parous present a higher number of strongly positive correlations, whilst nulliparous of strongly negative correlations. To obtain more detailed information on changes in correlations from nulliparous to parous, we run ForceAtlas2 and Fruchterman-Reingold algorithms implemented in Gephi software^67^. A specific network for each considered range of correlations is obtained, which can be numerically and graphically characterized for each group of women. For correlation networks different from those with extreme values (in bold, Table 1), the number of links is so high that the graphical representation does not provide useful information. For that reason, we only represent those networks with extreme correlation values (Fig. 4a–d).

The visual representation of the high positive correlation networks shows the existence of a reduced number of well-defined and separated clusters. This opens up the possibility to study the characteristics of those clusters and to identify the arising differences between nulliparous and parous. In this respect, and consistent with the results obtained for the whole networks, after computing the graph-theory parameters for the main cluster in each group, no significant changes have been found except for the node degree depending on parity.

**Figure 5:**
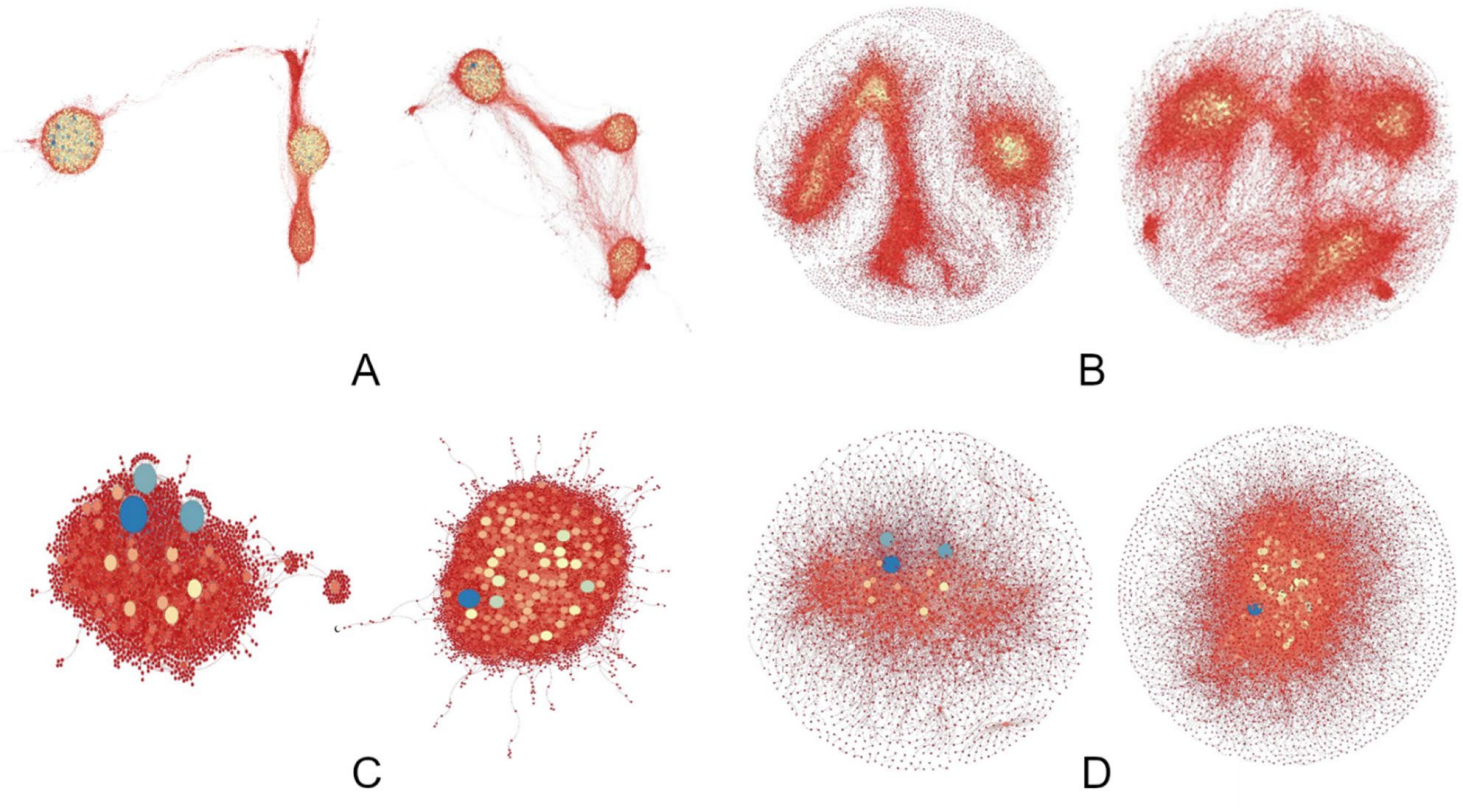
High-correlation specific networks with non-shared-nodes, ForceAtlas2 (**a**,**c**) and Frutcherman-Reingold (**b**,**d**) algorithms. For each figure, the network on the left shows the high correlation network specific to parous, while the network on the right shows the network specific to nulliparous. These are networks specific to a group, coherent with changes in the correlation structure after FFTP caused by variation in chromatin remodeling. (a, b) High positive correlation networks, 0.8 ≤ *ρ* < 1, ForceAtlas2 (a), Fruchterman-Reingold (b). (c, d) High negative correlation networks, -1 < *ρ* ≤ 0.8, ForceAtlas2 (c), Frutcherman-Reingold (d).

Fruchterman-Reingold^68^ and ForceAtlas2^69^ algorithms provide complementary representations of the considered network, visualizing the correlations between probes by mirroring physical attraction and repulsion forces. Whereas the Fruchterman-Reingold algorithm only takes into account the distance between two connected nodes to set the attraction–repulsion forces, ForceAtlas2 also considers the node degree, which facilitates the visualization of clusters^69^. We also modify this algorithm by modulating the size and assigned color of the represented probe depending on the number of probes presenting correlations with it. The results are the shapes and links depicted in Figs. 4 and 5. In both algorithms, different network shapes and/or probe sizes and colors between nulliparous and parous are the result of distinct correlation structures between groups. This is what happens, so this graphic evidence is coherent with the existence of changes in gene expressions and gene links after FFTP which are caused by the variation in chromatin remodeling. We can also numerically summarize these differences through several GT parameters^70–73^, collected in Table 2. The parameter “number of links” measures the volume/size of the network. For each probe/node, the “node degree” provides information on the connectedness of the considered probe. The “average node degree” measures the density of the network. The “average clustering coefficient” measures the presence of clusters inside the network, and “modularity” provides information on how different clusters inside the network are separated from each other. The “characteristic path length” measures the proximity between the probes in the network, and “diameter” refers to the compactness of the chains between probes. The “eigenvector centrality” measures the average influence of a probe, that is, its connectedness to other important/highly connected genes.

**Table 2:** Graph-theory parameters associated with the high-correlation networks. Those values of graph characteristics for which significant differences between nulliparous and parous were found have been marked with an asterisk.

Feature	Graph parameter	High positive correlation networks			High negative correlation networks		
		Parous	Nulliparous	*p *value	Parous	Nulliparous	*p *value
Basic	Number of links	244,854	208,870	NA	5946	17,095	NA
	Average node degree	58.31*	42.08*	*p* < 0.05	7.70*	12.60*	*p* < 0.05
Integration	Characteristic path length	7.10	6.47	NA	4.18	4.21	NA
	Diameter	21	22	NA	11	14	NA
Segregation	Average clustering coefficient	0.60*	0.50*	*p* < 0.05	0.00	0.00	*p* > 0.05
	Modularity	0.65	0.65	*p* > 0.05	0.45*	0.41*	*p* < 0.05
Centrality	Eigenvector centrality	0.28	0.29	*p* > 0.05	0.02*	0.04*	*p* < 0.05

As Table 2 summarizes, GT parameters agree with the existence of a genomic imprint for parous characterized by substantial changes in the way genes are interrelated. Concerning high positive correlations, for parous, this network has higher size, density, compactness and connectedness than the analogous network for nulliparous. When the high negative correlation networks are considered, we obtain the opposite result: the network for parous has lower volume, density, connectedness and compactness than that for nulliparous.

The examination of networks composed by non-shared nodes is also useful for our purpose. The correlation networks show the probes (nodes) with the specified correlation strength in their expression. We can then compare the resulting networks of each group in the same interval of correlation values, and remove those nodes that appear in the two networks, thus obtaining networks composed by non-shared nodes. These networks are relevant to reinforcing (or not) the hypothesis of a differentiated genetic signature in parous women. Thus, if these networks composed by non-shared nodes are networks with few nodes and slightly connected between them (compared to networks without ruling out any node), it would mean that the genetic signature is similar in both groups. On the contrary, a high percentage of genes appearing in these networks would provide additional evidence reinforcing the abovementioned hypothesis (a well-differentiated genetic signature in parous women). In this respect, for each range of correlation values, we observe that networks for parous are substantially composed by different probes and genes from those for nulliparous, an additional sign of the major scale of the redefinition of the relationships between genes caused by FFTP. These results show the extraordinary weight of the specificity of parous networks, to the point that 62% of the correlations are exclusive to parity (see Table S2 for further details). Figure 4 graphically shows these important specific networks for correlations higher than 0.8 in absolute value. After an inspection of the GT parameters for the specific networks, summarized in Table 3, we confirm the relevance of this restructuration of gene expression levels and relationships from a new perspective. If we compare parameters in Table 3 (see also Tables S3–S6 for the nodes with higher degrees in each network), we conclude that, from nulliparous to parous, the probes undergoing the greatest changes in the way they are expressed and linked are those involved in nulliparous specific networks. These probes are, precisely, the most numerous (52.8% for high positive correlations and 95% for high negative) and the most differently related with respect to parous.

**Table 3:** Graph-theory parameters associated with the high-correlation specific networks (specific-networks: non-shared nodes). Those values of graph characteristics for which significant differences between nulliparous and parous were found have been marked with an asterisk.

Feature	Graph parameter	High positive correlation networks			High negative correlation networks		
		Parous	Nulliparous	*p*-value	Parous	Nulliparous	*p*-value
Basic	Number of links	146,223	110,239	NA	5147	16,296	NA
	Average node degree	44.08*	25.38*	*p* < 0.05	6.86*	12.09*	*p* < 0.05
Integration	Characteristic path length	8.27	6.67	NA	4.27	4.23	NA
	Diameter	24	23	NA	12	14	NA
Segregation	Average clustering coefficient	0.31*	0.27*	*p* < 0.05	0.00	0.00	*p* > 0.05
	Modularity	0.65*	0.71*	*p* < 0.05	0.47*	0.41*	*p* < 0.05
Centrality	Eigenvector centrality	0.23	0.41	*p* < 0.05	0.02	0.04	*p* < 0.05

**Figure 6:**
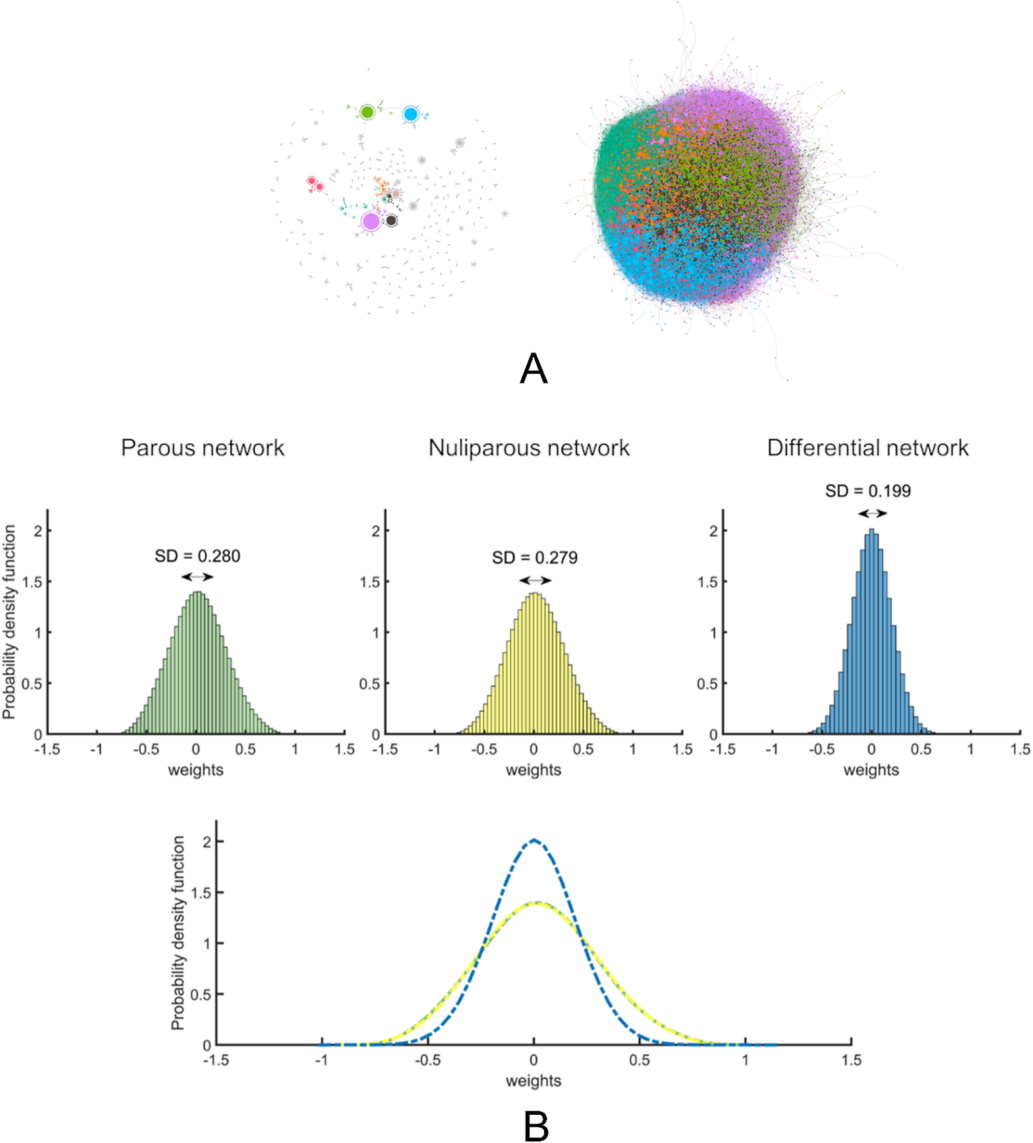
(**a**) Differential networks obtained from the direct comparison of parous and nulliparous groups depicted by ForceAtlas2 algorithm. For the figure on the left, the threshold of significance was set to α = 0.001, whereas α = 10^−6^ for the network on the right. Different colors were assigned according to the cluster class obtained by the modularity measure (see Methods). The node size is proportional to the node degree (see Methods). (**b**): Probability density function (PDF) of the parous, nulliparous and differential networks. Parous and nulliparous networks show very similar distributions of the correlation weights. The differential network, however, is more compactly distributed despite having a larger range of possible values (from -2 to 2). The three PDFs are shown superimposed at the bottom, highlighting the greater compactness of the differential network (in blue). Values at the extremes of the differential network correspond to the connections (correlations) with highly significant differences between parous and nulliparous networks.

The wide redefinition of gene interrelationships after FFTP is also patent when changes in correlations are inspected. The identification of networks formed by the same probes in nulliparous and parous, and which significantly and homogeneously change their mutual correlations, reveals the existence of gene structures modifying their behavior as a whole after FFTP. At α = 0.05, the networks with significantly different correlation links between nulliparous and parous represent 53.3% of the total number. In this respect, it is worth noting three questions. First, these changes in correlations need not be accompanied by changes in mean expression levels of the probes involved: About 746,000 pairs of probes present a significant modification in correlations, but not in their expression values. Second, the networks arising from modifications in the correlation coefficients are formed by the same genes/probes in both groups, with correlations between them taking any value, since the networks are defined by the correlation changes, but not by the correlation values. This means that these networks and these changes are different from those identified by usual DCNA, which is based on imposing a value for the correlation linking the probes. Figure 6a shows these networks defined by correlation changes and their structures for two different significance levels (α = 0.01 and α = 10^−6^). And third, from nulliparous to parous, the networks/modules changing the correlations linking their constituent probes/genes are affected as a whole. This suggests the existence of significant clusters, formed by genes heterogeneously correlated, but homogeneously changing these correlations from nulliparous to parous. This feature of the FFTP genomic signature arises from the study of the probability density function (PDF) of the correlation values for parous, nulliparous, and the differential networks. As depicted in Fig. 6b, networks obtained from the direct subtraction of parous from nulliparous are more compactly distributed. Indeed, despite having a range of possible values between -2 and 2, the PDF of the subtraction networks presents a much lower standard deviation than those for parous and nulliparous. This result appears only when the covariance between parous and nulliparous correlations is highly positive, thus confirming the existence of changes in correlations associated to FFTP affecting clusters of probes/genes as a whole, independently of how the involved probes/genes are correlated to each other inside the cluster. In short, these facts suggest that the chromatin remodeling associated to FFTP is a far-reaching phenomenon, implying changes at the individual gene level that coexist with, and are independent of, changes at the joint level affecting clusters of genes in several dimensions.

The multidimensionality of FFTP induced changes is even clearer when the number of considered probes is greater than two. Since the computational requirements to explore all the possibilities are enormous, we present an illustrative extrapolation of our findings for a reduced subset of probes. By designing differential MRA computational procedures, we conclude that there are about 900 million significant stable relationships linking 4 genes specific to parous (66 million to nulliparous), with a very intricate characterization: at least one of the involved genes changes does not change its average expression level, not all are in the same correlation network, and not all homogenously modify their correlations. This has important implications, since this method identifies small-scale systems of self-regulated processes inherent to parity, and opens up the identification of hub genes and modules with genes that are not all highly correlated or homogeneously affected, and that could be biologically meaningful^74–78^. It is worth noting that these numbers suggest again higher complexity, connectedness and compactness of the genomic profile after FFTP.

Our conclusions after applying these new analysis criteria from GT, IT, DCNA, and differential MRA, confirm the existence of a specific genomic signature induced by FFTP, centered on a variation of chromatin remodeling, and characterized by the coexistence and independence of changes at the gene individual level and at the joint cluster level, which also encompass various dimensions. Our approaches are able to disentangle and identify these complex genomic changes, allowing potential meaningful new pathways, hubs and modules to be identified and reducing costly lab work.

## Methods

### Gene-by-gene analysis

#### Mean value

For each probe, the arithmetic mean of its expression level is calculated in both groups. These two values for each probe are then compared by running a two-sample t-test for equal means (two-sided, non-equal variances). At the significance level α = 0.05, and taking as reference the nulliparous group, the sample size allows a difference of means of 10% between groups to be detected for 98.98% of probes at 90% of statistical power. For the remaining 1.02% of probes, the test detects differences of means in the interval (10%, 31%]. Appropriate algorithms for all these computations were written and run in R3.6.2. Changes in mean values are also interpreted by applying cluster analysis.

#### Area under the cumulative distribution function

For each probe, the empirical cumulative distribution functions of the statistical series defined by the 42 expression level data for nulliparous and the 71 for parous are calculated. For each probe, once the area under each cumulative distribution function has been computed, we calculate the difference between nulliparous and parous. Appropriate algorithms for these computations were written and run in Matlab. In addition, to check equality of statistical distributions, non-parametric two-sided Kolmogorov–Smirnov and Wilcoxon Rank Sum tests at significance level α = 0.05 were run in R3.6.2. Given that the evidence on the reliability of the use of FDR control in the Kolmogorov–Smirnov and Wilcoxon tests is not clear^56–58^, we present both the analyses in terms of *p *values and adjusted *p *values. In addition, for these non-parametric tests, the statistical power must be estimated by simulation, and it is very sensitive to the parameters and distributions of the data series for each probe, as well as to the number of simulations. This requires the previous characterization of the statistical distribution of the 18,653 probes for the two groups, a process that consumes an enormous amount of time and huge computer resources with no profitability from the research perspective. For illustrative purposes, after checking for normality (99.9% of the series, Shapiro test, α = 0.05) and taking as reference the average value of the difference in means and standard deviations, the statistical power of the Wilcoxon Rank sum test is 0.73 (algorithm written and run in R3.6.2, 100,000 simulations).

#### Shannon entropy

Since the objective is to compare the entropy for each probe between nulliparous and parous, equal indeterminacy degree in both groups must be assumed by defining the same set of possible outcomes. Once the package “entropy” is installed in R3.6.2, this is done by applying the function *discretize*. After computing the maximum and minimum expression level for each probe in the whole data set (nulliparous plus parous), a common grid of intervals is considered, and the subsequent histogram computed through this command. Then, for each probe, the Shannon entropy of the statistical series defined by the expression level data for nulliparous and parous can be separately calculated making use of the *entropy* function (algorithm in R3.6.2). For each probe and group, 1,000 resamples were randomly selected, and their SE calculated (bootstrapping). After computing the mean for each probe in the different groups, a t-test was run to conclude statistically significant differences (FDR control with BH method, adjusted *p *value < 0.05).

From the two *mean* Shannon entropies obtained for each probe, we run a cluster analysis, represented in Fig. 2.

#### Cluster analysis and dendrograms (Fig. 2a,b)

Once the mean value of the expression level and the Shannon entropy of each probe for each group have been obtained, we carry out a cluster analysis for each of these two measures. In addition, a dendrogram representation is provided. We use the Euclidean measure to obtain the distance matrix, and the complete agglomeration method for clustering. For each variable, two heatmaps are presented. The left-hand heatmap considers raw-data and clusterizes according to similarity of values between groups (red to green: low to high values). With the same reordering, the right-hand heatmap shows differences after computing the row-Z-score. Cluster analysis and dendrograms were obtained by writing algorithms in R3.6.2.

#### Representation of changes in genomic structure (Fig. 3a–e)

For each of the considered measures (ME, CD and SE), the representation of the structural change in genomic expression has been obtained following the same procedure. First, for each probe and each group, the value of the considered variable (ME, CD or SE) is computed. After normalizing the value for each probe in nulliparous to the unity (horizontal lines in blue), the relative value of the probes for parous are calculated. Finally, the relative values for parous are ordered from highest to lowest and represented (curves in green) to show the structural changes associated to FFTP. This normalization allows the correlation between criteria to be better visualized. For each pair of criteria, the simultaneous relative changes are depicted after ordering with respect to one of the criteria.

#### Gene ontology

Gene ontology (GO) analysis was implemented making use of the Gene Ontology Consortium computational models^79–81^ (release 2020–11-17: 44.167, PANTHER classification system, adjusted *p *value < 0.05).

#### Extrapolations

We conclude that there exist about 746,000 pairs of probes with a significant modification in correlations, but not in their expression values. To obtain this number, we wrote an algorithm in R3.6.2 to select pairs of genes whose mean expression level do not change between groups (two-sample t-test for equal means, two-sided and non-equal variances, significance level α = 0.05), but whose correlation, on the contrary, significantly changes (test based on Fisher r-to-z transformation, significance level α = 0.05). Applying this algorithm to the original dataset for 18,653 probes is very demanding in terms of time and computing memory, so we ran the procedure over a subset of 3000 randomly selected probes and extrapolated the results over the total set of 18,653 probes.

#### Differential multiple regression analysis

Differential Multiple Regression Analysis was applied to detect evidence on the existence of steady strong relationships between 4 genes specific to nulliparous and to parous, and which present additional features with interest for our research. We start our study with those pairs of probes presenting a correlation with abs(ρ) > 0.8 for a group, and changing more than 0.4 their correlation between groups (1728 pairs of probes). This initial set of probe pairs is divided into 4 different groups, respectively A, B, C and D: ρP > 0.8 and ρN < 0.4 (A); ρN > 0.8 and ρP < 0.4 (B); ρP < -0.8 and ρN > -0.4 (C); and ρN < -0.8 and ρP > -0.4 (D), where ρP and ρN denote, respectively, the correlation coefficient in parous and nulliparous. There are 716 pairs in A, 740 in B, 61 in C and 211 in D. Assuming a linear relationship, a third and fourth probe are added to run OLS. Due to the prohibitive computational costs, this is done over a limited subset of probes randomly selected. First, for each subset (A), (B), (C) and (D), a reduced number of pairs is randomly selected. More specifically, we have checked 3 pairs in group A; 4 pairs in B; 4 pairs in C; and 4 pairs in D. Then OLS multiple regressions are run according to the following procedure: the first probe in the pair is considered as the regressand, while the second as the first regressor; then a third probe randomly selected is added as the second regressor; the third regressor being, consecutively, any of the remaining 18,651 probes. Following this protocol, 412,146,871 linear relationships have been computed (0.14% of the total number), distributed as follows: 49% in A; 16.2% in B; 15.3% in C; and 19.5% in D. From these OLS regressions, we selected those verifying the following conditions: the coefficient of determination R2 changes between groups from [1,0.8) to (0.4,0]; at least one of the involved genes does not change its average expression level; not all the genes in the regression are in the same correlation network; and not all homogenously modify their correlations. We thus identify stable relationships between genes in a group that disappear in the other group, and that, in addition, can not be identified through any of the other considered gene-by-gene or gene-to-gene criteria. For the selected subsets of probes, there are 3,821,047 (1.86% of the analyzed number) of the said relationships in the first group, 326,084 (0.66%) in the second, 48,683 (0.07%) in the third and 116,692 (0.15%) in the fourth. An extrapolation to the total number of pairs in each group implies around 900 million of stable relationships of this type for parous disappearing for nulliparous, and 66 million for nulliparous disappearing for parous. All computations were run in R3.6.2.

### Network analysis

This analysis, based on weighted correlation networks, is usually known as Differential Co-expression Network Analysis (DCNA) when applied to the genetic field. DCNA is a data mining method based on presenting variables as nodes (translated as gene expression in genetics) and pairwise correlations between them as links in the network. Due to the versatility of this method and its utility for visualizing and recognizing patterns in complex systems, it has been exported to several different areas apart from genetics, such as psychiatry^82^, neuroscience^83^ or plant ecology^84^.

#### Gene-to-gene network generation

The levels of genetic expression were used to calculate Pearson’s rank correlations for all probe pairs using the Matlab ‘Statistics and Machine Learning Toolbox’, independently for parous and nulliparous groups. Due to the large amount of data considering all the possible probe pairings (18,853 genes result in more than 177 billion correlations), a thresholding in steps of 0.2 was performed on the two correlation matrices generated. This gives rise to a set of networks for parous and nulliparous groups: 9 networks for each group (see Table S2). From them, we define “high correlation networks” as those with correlations greater than 0.8 in absolute value (see Fig. 4). For a significance level α = 0.05 and a statistical power 0.8, the critical values to conclude significant correlation coefficients for nulliparous and parous are, respectively, ± 0.37 and ± 0.29.

#### Selection of non-shared nodes

We compare the resulting networks of each group in the same interval of correlation values, and remove those nodes that appear in the two networks, thus obtaining networks composed by non-shared nodes.

#### Network visualization

Gephi is an open source software for network analysis^67^. We used the updated version of the software 0.9.2, which includes several tools and customizable packages for network visualization and graph parameter computation. Among the multiple features of this software, it is worth mentioning the force-based algorithms that allow us an easy visualization of complex patterns. For this work, we used the well-known Fruchterman-Rheingold algorithm^68^ that uses custom forces of attraction and repulsion dependent on the distance between nodes. In particular, this algorithm establishes a square proportionality between the distance and the force (as for electromagnetic attraction) and a linear proportionality between the distance and the force (as for spring repulsion). In principle, all nodes/probes are equally affected by repulsion and attraction forces. To determine the position, the Frutcherman-Reingold algorithm does not take into account the node degree (the number of probes correlated with it at the specified threshold), which graphically only defines the size of the node. Therefore, the position of each node only depends on the distance to other nodes and on the specific nodes with which they are connected.

Additionally, the ForceAtlas2 algorithm^69^ was also applied to the resulting networks as a complementary approach for visualization, which considers both the distance and the node degree of the connected nodes^69^. This relation with the node degree is codified in the repulsion force by setting it as proportional to the product of the degrees plus one^68^. Thus, while the Fruchterman-Rheingold algorithm takes into account the topology of the network to place the nodes, the ForceAtlas2 algorithm considers both the topology and node degree (the number of nodes correlated to it at the considered threshold) of each of the nodes in the network. These two methods are not deterministic, and the coordinates of each point do not reflect any specific variable, so the position of a node/probe must be interpreted as compared to the others. These force-based algorithms have an important advantage: they turn structural proximities, such as those in communities or genetic clusters^85^, into visual proximities, informing on the underlying structure.

Color and size filters were incorporated into the visualization of the networks to enhance the recognition of patterns and communities in genetic networks. In this way, the size and color of each node are directly related to the degree of the node (see next section for additional details on node degree).

#### Graph theory parameters

With the aim of providing objective and quantifiable measures to characterize the obtained networks, we rely on the Graph Theory field. Thus, complementary parameters were selected to provide a comprehensive view of the difference in the graph structure of the networks derived from the parous and nulliparous groups. We have classified these parameters into four groups of features^70–73,86–88^: basic, integration, segregation and centrality.

#### Basic features

- The number of links is the simplest measure useful for quantifying the volume/size of the network.
- Node degree is the number of edges connected to the node. When averaging, it therefore measures the degree of global connectedness or density of the network. It is strongly related to the total number of links of the network, but also considering the number of nodes.

#### Integration features

- Characteristic path length is the average shortest path length between all pairs of nodes in the network^70,71^.
- The graph diameter is the largest number of vertices which must be traversed to connect any combination of node pairs, excluding backtracks, detours, or loops.

Due to the nature of our data, the algorithm proposed by Brandes^86^ was followed for the computation of the integration features.

#### Segregation features

- The clustering coefficient is a measure of network segregation defined as the ratio between the number of closed triangles and open triplex in a particular node. This is equivalent to the fraction of the node's neighbors that are also neighbors of each other^71^. For large graphs, as in this case, optimized algorithms are needed to reduce computational cost. In this study, we used the solution proposed by Latapy^87^ directly implemented in Gephi.
- Modularity measures the strength of division of a network into modules (usually called communities), determining when there are more links within such modules than expected on the basis of chance. The method for large communities proposed by Blondel and colleagues^72^, directly implemented in Gephi, was applied.

#### Centrality features

- Eigenvector centrality quantifies the influence of a node on a network^88^. It is based on the concept that connections to other well-connected (high-scoring) nodes must contribute more to the final centrality score than connections with nodes with low relevancy in the network (with low-scoring).

#### Statistics for graph parameters

In order to provide measurable and statistic evidence for the graph parameter differences of the networks, the following procedure was performed:

1. 90% of the nodes were randomly selected from each network.
2. The graph parameters were computed in the new networks.
3. This procedure was repeated 100 times.
4. Statistical differences between both distributions were analyzed using a Student’s t-test (*α* = 0.05).

Due to the high computational cost, this procedure is feasible in all graph measurements except those of integration (characteristic path length and diameter).

#### Considerations about the threshold used in the networks

In this study, the threshold used for the network representation was based on extreme values of the Pearson’s rho coefficient (ρ = 0.9). A theoretically better alternative to this choice would be to control the correlations for false discovery rate (FDR) and select only those with an adequate significance threshold. However, FDR requires the *p *values to be ordered, with a very high computational cost due to the great dimensionality of the problem (around 174 million different correlations). That is why we decided to use a threshold based on the ρ value. The consideration of this extreme value for ρ = 0.9 minimizes this problem associated to false discovery.

## Supplementary Information

Supplementary Information.
