# Supplementary material for: The role of gene to gene interaction in the breast’s genomic signature of pregnancy
Source: Sci Rep. 2021 Jan 29;11:2643. doi: 10.1038/s41598-021-81704-8 (PMC7846553; doi:10.1038/s41598-021-81704-8)
Supplement: Supplementary file 1 — Supplementary Information. [file 41598_2021_81704_MOESM1_ESM.docx]

Supplementary material for

**The role of gene to gene interaction in the breast’s genomic signature of pregnancy**

**Authors**

**Pedro J. Gutiérrez-Díez, Javier Gomez-Pilar, Roberto Hornero, Julia Martínez-Rodríguez, Miguel A. López-Marcos, Jose Russo**

**This file includes:**

Table S1. Changes in genes associated to chromatin remodeling.

Table S2. Size of the networks for each threshold formed by nodes only in parous or only in nulliparous (specific-networks: non-shared nodes).

Table S3. Graph-theory measures applied to the 10 nodes with higher node degree in the parous networks (0.8 ≤ ρ < 1).

Table S4. Graph-theory measures for the 10 nodes with higher node degree in the nulliparous networks (0.8 ≤ ρ < 1).

Table S5. Graph-theory measures for the 10 nodes with higher node degree in the parous networks (-1< ρ ≤ -0.8).

Table S6. Graph-theory measures for the 10 nodes with higher node degree in the nulliparous networks (-1< ρ ≤ -0.8).

Table S7-S15. Gene Ontology (GO) analysis results for the two new applied criteria: CD and SE

1. Cumulative Distribution (CD) Criterion

S7. Biological processes

S8. Molecular Functions

S9. Cellular Components

1. Shannon Entropy (SE) criterion
   1. Higher SE in parous.

S10. Biological processes

S11. Molecular Functions

S12. Cellular Components

- 1. Lower SE in parous.

S13. Biological processes

S14. Molecular Functions

S15. Cellular Components

Table S1. Changes in genes associated to chromatin remodeling.

| Genes associated to chromatin remodeling | Probe | Change in expression level | Change in distribution | Change in Shannon Entropy |
| --- | --- | --- | --- | --- |
| APOBEC3G | 204205_at | U | Y | H+ |
| TOX | 204529_s_at |  |  |  |
|  | 204530_s_at |  |  | H++ |
| UHRF1 | 213118_at |  |  | H+ |
|  | 226135_at |  |  |  |
| NAP1L2 | 219368_at | U | Y | H+ |
| KDM4B | 212492_s_at |  |  |  |
|  | 212495_at | D | Y |  |
|  | 212496_s_at | D |  |  |
| TOX3 | 214774_x_at | D | Y |  |
|  | 215108_x_at | D | Y |  |
|  | 216623_x_at | D | Y |  |
| WIF1 | 204712_at |  |  |  |
| FOXQ1 | 227475_at |  |  |  |
| FZD8 | 224325_at |  |  | H++ |
|  | 227405_s_at | U | Y |  |
| SDC1 | 201286_at | U |  |  |
|  | 201287_s_at | U |  |  |
| EAF2 | 219551_at |  | Y |  |
| BHLHE22 | 228636_at |  |  |  |
| DSC3 | 206032_at | U | Y |  |
|  | 206033_s_at | U | Y |  |
| KRT5 | 201820_at | U | Y |  |
| EGR3 | 206115_at | U |  | H+ |
| RASGRP1 | 205590_at | U | Y |  |
| CBX3 | 1555920_at | U | Y |  |
| L3MBTL1 | 210306_at | U | Y |  |
| CHD1L | 212539_at |  |  |  |
|  | 238070_at |  |  | H+ |
| CHD1L | 235791_x_at |  | Y |  |
| CHD8 | 212571_at |  |  |  |
| CHD9 | 212615_at |  |  | L-- |
|  | 229586_at |  |  | L-- |
|  | 235388_at |  |  |  |
|  | 239654_at |  |  |  |
|  | 242157_at |  |  |  |
|  | 220586_at |  |  |  |
| CHD7 | 218829_s_at |  |  |  |
|  | 226123_at |  |  | L-- |
| CHD6 | 225026_at | U | Y |  |
|  | 225031_at |  |  |  |
| EZH2 | 203358_s_at | U |  |  |
| EZH1 | 239198_at |  |  |  |
|  | 203249_at |  |  |  |
|  | 32259_at |  |  |  |
| CHD3 | 208807_s_at | U | Y |  |
|  | 208806_at | U | Y | L-- |

**Change in expression level**: U and D denote, respectively, up and down regulation [two-Sample t-test, α=5%]. **Change in distribution**: Y indicates change in cumulative distribution Kolmogorov-Smirnov test, α=5%]. **Change in Shannon Entropy**: H++ and H+ denote, respectively, relative gain with respect to nulliparous in first quartile Q1 and second quartile Q2; L- - denotes relative loss with respect to nulliparous in quartile 1 Q1. Range of entropy relative increases: (1,1.58333]. Range of entropy relative decreases: (0.73005,1].

Table S2. Size of the networks for each threshold formed by nodes only in parous or only in nulliparous (specific-networks: non-shared nodes).

|  | Parous | | Nulliparous | |
| --- | --- | --- | --- | --- |
| Correlation **range** | # of correlations | % of correlations | # of correlations | % of correlations |
| **(0.8, 1]** | **146223** | **0.17** | **110239** | **0.16** |
| (0.6, 0.8] | 1931513 | 2.30 | 1771105 | 2.64 |
| (0.4, 0.6] | 7796114 | 9.27 | 7978531 | 11.90 |
| (0.2, 0.4] | 18691993 | 22.22 | 1899105 | 2.83 |
| [-0.2, 0.2] | 30521321 | 36.28 | 30413860 | 45.37 |
| [-0.4 -0.2) | 17310607 | 20.58 | 17535439 | 26.16 |
| [-0.6, -0.4) | 6751562 | 8.03 | 6253965 | 9.33 |
| [-0.8, -0.6) | 973242 | 1.16 | 1057234 | 1.58 |
| **[-1 -0.8)** | **5147** | **0.01** | **16296** | **0.02** |
| Total | 84127722 | 100 | 67035774 | 100 |

High-correlation networks are marked in bold

Table S3. Graph-theory measures applied to the 10 nodes with higher node degree in the parous networks (0.8 ≤ ρ < 1).

| Gene | Node degree | Clustering coefficient | Eigenvector centrality |
| --- | --- | --- | --- |
| 222985_at | 690 | 0.41 | 1 |
| 202620_s_at | 689 | 0.40 | 0.98 |
| 203282_at | 672 | 0.42 | 0.98 |
| 211432_s_at | 670 | 0.41 | 0.97 |
| 226834_at | 655 | 0.39 | 0.9q |
| 238003_at | 654 | 0.43 | 0.97 |
| 231683_at | 646 | 0.43 | 0.96 |
| 218665_at | 638 | 0.45 | 0.97 |
| 218736_s_at | 634 | 0.44 | 0.95 |
| 235129_at | 623 | 0.46 | 0.95 |

Table S4. Graph-theory measures applied to the 10 nodes with higher node degree in the nulliparous networks (0.8 ≤ ρ < 1).

| Gene | Node degree | Clustering coefficient | Eigenvector centrality |
| --- | --- | --- | --- |
| 202990_at | 582 | 0.36 | 0.993 |
| 209210_s_at | 577 | 0.34 | 0.947 |
| 210640_s_at | 569 | 0.38 | 1.0 |
| 226657_at | 562 | 0.38 | 0.985 |
| 216331_at | 551 | 0.39 | 0.981 |
| 211980_at | 548 | 0.38 | 0.947 |
| 228445_at | 543 | 0.38 | 0.943 |
| 226834_at | 537 | 0.39 | 0.947 |
| 212646_at | 536 | 0.38 | 0.923 |
| 212097_at | 536 | 0.383 | 0.934 |

Table S5. Graph-theory measures applied to the 10 nodes with higher node degree in the parous networks (-1< ρ ≤ -0.8).

| Gene | Node degree | Clustering coefficient | Eigenvector centrality |
| --- | --- | --- | --- |
| 212727_at | 323 | 0.00 | 1.0 |
| 218816_at | 258 | 0.00 | 0.88 |
| 227210_at | 212 | 0.00 | 0.61 |
| 202193_at | 141 | 0.00 | 0.49 |
| 200944_s_at | 114 | 0.00 | 0.43 |
| 212599_at | 110 | 0.00 | 0.41 |
| 242354_at | 108 | 0.00 | 0.39 |
| 225792_at | 107 | 0.00 | 0.50 |
| 203395_s_at | 99 | 0.00 | 0.43 |
| 203124_s_at | 95 | 0.00 | 0.36 |

Table S6. Graph-theory measures applied to the 10 nodes with higher node degree in the nulliparous networks (-1< ρ ≤ -0.8).

| Gene | Node degree | Clustering coefficient | Eigenvector centrality |
| --- | --- | --- | --- |
| 208837_at | 305 | 0.00 | 1.00 |
| 201479_at | 206 | 0.00 | 0.77 |
| 202489_s_at | 195 | 0.00 | 0.77 |
| 218035_s_at | 178 | 0.00 | 0.76 |
| 242354_at | 175 | 0.00 | 0.82 |
| 212727_at | 167 | 0.00 | 0.60 |
| 225846_at | 167 | 0.00 | 0.84 |
| 213285_at | 160 | 0.00 | 0.78 |
| 202546_at | 154 | 0.00 | 0.52 |
| 201478_s_at | 150 | 0.00 | 0.65 |

GENE ONTOLOGY ANALYSIS RESULTS

Analysis Type: PANTHER Overrepresentation Test. Adjusted *p*-value<0.05

1. **CUMULATIVE DISTRIBUTION (CD) CRITERION**

Considered set: 1986 probes changing their statistical distribution from nulliparous to parous, Kolmogorov-Smirnov and Wilcoxon tests, adjusted *p*-value<0.05

S7. BIOLOGICAL PROCESSES

| \|  \| Homo sapiens \| RESULTS \| \| \| \| \| \| \| --- \| --- \| --- \| --- \| --- \| --- \| --- \| --- \| \| GO biological process complete \| # \| # \| expected \| Fold Enrichment \| +/- \| raw P value \| FDR \| \| mRNA splice site selection \| 31 \| 11 \| 1.96 \| 5.62 \| + \| 2.42E-05 \| 5.33E-03 \| \| cellular component biogenesis \| 2654 \| 232 \| 167.63 \| 1.38 \| + \| 9.08E-07 \| 3.06E-04 \| \| cellular component organization or biogenesis \| 5922 \| 488 \| 374.05 \| 1.30 \| + \| 5.15E-11 \| 2.04E-07 \| \| cellular process \| 15598 \| 1063 \| 985.21 \| 1.08 \| + \| 9.26E-07 \| 3.06E-04 \| \| cellular component organization \| 5703 \| 472 \| 360.22 \| 1.31 \| + \| 7.37E-11 \| 2.34E-07 \| \| cellular component assembly \| 2394 \| 215 \| 151.21 \| 1.42 \| + \| 4.58E-07 \| 1.86E-04 \| \| spliceosomal complex assembly \| 56 \| 13 \| 3.54 \| 3.68 \| + \| 1.85E-04 \| 2.77E-02 \| \| mRNA splicing, via spliceosome \| 304 \| 41 \| 19.20 \| 2.14 \| + \| 2.56E-05 \| 5.56E-03 \| \| mRNA processing \| 489 \| 67 \| 30.89 \| 2.17 \| + \| 4.73E-08 \| 2.68E-05 \| \| RNA processing \| 929 \| 99 \| 58.68 \| 1.69 \| + \| 1.91E-06 \| 5.61E-04 \| \| gene expression \| 2105 \| 179 \| 132.96 \| 1.35 \| + \| 1.01E-04 \| 1.80E-02 \| \| macromolecule metabolic process \| 6333 \| 512 \| 400.01 \| 1.28 \| + \| 2.15E-10 \| 4.27E-07 \| \| organic substance metabolic process \| 8011 \| 597 \| 505.99 \| 1.18 \| + \| 8.10E-07 \| 2.86E-04 \| \| metabolic process \| 8592 \| 636 \| 542.69 \| 1.17 \| + \| 5.14E-07 \| 2.04E-04 \| \| nucleic acid metabolic process \| 2241 \| 189 \| 141.55 \| 1.34 \| + \| 8.91E-05 \| 1.66E-02 \| \| cellular metabolic process \| 7784 \| 587 \| 491.66 \| 1.19 \| + \| 2.05E-07 \| 9.58E-05 \| \| primary metabolic process \| 7578 \| 567 \| 478.64 \| 1.18 \| + \| 1.31E-06 \| 4.06E-04 \| \| nitrogen compound metabolic process \| 7091 \| 539 \| 447.88 \| 1.20 \| + \| 4.19E-07 \| 1.75E-04 \| \| mRNA metabolic process \| 700 \| 76 \| 44.21 \| 1.72 \| + \| 1.83E-05 \| 4.15E-03 \| \| RNA splicing, via transesterification reactions with bulged adenosine as nucleophile \| 304 \| 41 \| 19.20 \| 2.14 \| + \| 2.56E-05 \| 5.48E-03 \| \| RNA splicing, via transesterification reactions \| 307 \| 42 \| 19.39 \| 2.17 \| + \| 1.65E-05 \| 3.85E-03 \| \| RNA splicing \| 410 \| 60 \| 25.90 \| 2.32 \| + \| 2.79E-08 \| 1.64E-05 \| \| intraciliary transport involved in cilium assembly \| 40 \| 12 \| 2.53 \| 4.75 \| + \| 4.13E-05 \| 8.40E-03 \| \| cilium assembly \| 359 \| 55 \| 22.68 \| 2.43 \| + \| 2.15E-08 \| 1.37E-05 \| \| organelle assembly \| 773 \| 82 \| 48.82 \| 1.68 \| + \| 1.69E-05 \| 3.87E-03 \| \| organelle organization \| 3586 \| 311 \| 226.50 \| 1.37 \| + \| 1.03E-08 \| 7.10E-06 \| \| plasma membrane bounded cell projection assembly \| 453 \| 66 \| 28.61 \| 2.31 \| + \| 4.90E-09 \| 3.89E-06 \| \| cell projection assembly \| 470 \| 67 \| 29.69 \| 2.26 \| + \| 8.73E-09 \| 6.29E-06 \| \| cell projection organization \| 1200 \| 111 \| 75.79 \| 1.46 \| + \| 1.42E-04 \| 2.29E-02 \| \| plasma membrane bounded cell projection organization \| 1154 \| 107 \| 72.89 \| 1.47 \| + \| 1.75E-04 \| 2.67E-02 \| \| cilium organization \| 382 \| 58 \| 24.13 \| 2.40 \| + \| 1.65E-08 \| 1.09E-05 \| \| microtubule-based process \| 814 \| 83 \| 51.41 \| 1.61 \| + \| 6.59E-05 \| 1.31E-02 \| \| morphogenesis of a polarized epithelium \| 47 \| 13 \| 2.97 \| 4.38 \| + \| 4.07E-05 \| 8.39E-03 \| \| non-motile cilium assembly \| 49 \| 12 \| 3.09 \| 3.88 \| + \| 2.12E-04 \| 3.12E-02 \| \| regulation of alternative mRNA splicing, via spliceosome \| 77 \| 16 \| 4.86 \| 3.29 \| + \| 1.09E-04 \| 1.88E-02 \| \| regulation of mRNA splicing, via spliceosome \| 115 \| 24 \| 7.26 \| 3.30 \| + \| 2.20E-06 \| 6.33E-04 \| \| regulation of mRNA processing \| 154 \| 31 \| 9.73 \| 3.19 \| + \| 1.54E-07 \| 7.43E-05 \| \| regulation of mRNA metabolic process \| 352 \| 43 \| 22.23 \| 1.93 \| + \| 1.39E-04 \| 2.30E-02 \| \| regulation of RNA metabolic process \| 3801 \| 324 \| 240.08 \| 1.35 \| + \| 2.64E-08 \| 1.61E-05 \| \| regulation of macromolecule metabolic process \| 6491 \| 515 \| 409.99 \| 1.26 \| + \| 2.88E-09 \| 3.26E-06 \| \| regulation of metabolic process \| 7025 \| 556 \| 443.72 \| 1.25 \| + \| 5.18E-10 \| 6.84E-07 \| \| regulation of biological process \| 11960 \| 863 \| 755.42 \| 1.14 \| + \| 4.88E-09 \| 4.08E-06 \| \| biological regulation \| 12645 \| 914 \| 798.69 \| 1.14 \| + \| 1.58E-10 \| 3.58E-07 \| \| regulation of nucleobase-containing compound metabolic process \| 4073 \| 336 \| 257.26 \| 1.31 \| + \| 3.38E-07 \| 1.45E-04 \| \| regulation of nitrogen compound metabolic process \| 5895 \| 472 \| 372.34 \| 1.27 \| + \| 8.50E-09 \| 6.42E-06 \| \| regulation of cellular metabolic process \| 6304 \| 508 \| 398.18 \| 1.28 \| + \| 4.52E-10 \| 6.53E-07 \| \| regulation of cellular process \| 11395 \| 836 \| 719.74 \| 1.16 \| + \| 3.62E-10 \| 5.75E-07 \| \| regulation of primary metabolic process \| 6086 \| 488 \| 384.41 \| 1.27 \| + \| 3.15E-09 \| 3.34E-06 \| \| regulation of RNA splicing \| 158 \| 30 \| 9.98 \| 3.01 \| + \| 7.18E-07 \| 2.71E-04 \| \| regulation of gene expression \| 4894 \| 394 \| 309.12 \| 1.27 \| + \| 2.25E-07 \| 1.02E-04 \| \| G2/M transition of mitotic cell cycle \| 142 \| 22 \| 8.97 \| 2.45 \| + \| 3.33E-04 \| 4.56E-02 \| \| cell cycle G2/M phase transition \| 144 \| 23 \| 9.10 \| 2.53 \| + \| 1.79E-04 \| 2.71E-02 \| \| cell cycle phase transition \| 298 \| 40 \| 18.82 \| 2.13 \| + \| 3.57E-05 \| 7.45E-03 \| \| cell cycle process \| 1059 \| 110 \| 66.89 \| 1.64 \| + \| 1.59E-06 \| 4.76E-04 \| \| cell cycle \| 1383 \| 137 \| 87.35 \| 1.57 \| + \| 8.76E-07 \| 3.02E-04 \| \| mitotic cell cycle phase transition \| 290 \| 38 \| 18.32 \| 2.07 \| + \| 8.11E-05 \| 1.57E-02 \| \| mitotic cell cycle process \| 680 \| 77 \| 42.95 \| 1.79 \| + \| 4.62E-06 \| 1.29E-03 \| \| mitotic cell cycle \| 757 \| 86 \| 47.81 \| 1.80 \| + \| 1.09E-06 \| 3.52E-04 \| \| mitotic cell cycle checkpoint \| 159 \| 24 \| 10.04 \| 2.39 \| + \| 2.37E-04 \| 3.39E-02 \| \| negative regulation of mitotic cell cycle \| 303 \| 42 \| 19.14 \| 2.19 \| + \| 9.48E-06 \| 2.43E-03 \| \| regulation of mitotic cell cycle \| 597 \| 72 \| 37.71 \| 1.91 \| + \| 1.09E-06 \| 3.46E-04 \| \| regulation of cell cycle \| 1209 \| 133 \| 76.36 \| 1.74 \| + \| 4.02E-09 \| 3.55E-06 \| \| negative regulation of cell cycle \| 577 \| 71 \| 36.44 \| 1.95 \| + \| 7.17E-07 \| 2.78E-04 \| \| negative regulation of cellular process \| 4952 \| 400 \| 312.78 \| 1.28 \| + \| 1.03E-07 \| 5.29E-05 \| \| negative regulation of biological process \| 5592 \| 435 \| 353.20 \| 1.23 \| + \| 1.38E-06 \| 4.22E-04 \| \| cell cycle checkpoint \| 199 \| 29 \| 12.57 \| 2.31 \| + \| 1.04E-04 \| 1.81E-02 \| \| regulation of gene expression, epigenetic \| 172 \| 25 \| 10.86 \| 2.30 \| + \| 3.54E-04 \| 4.76E-02 \| \| negative regulation of mitotic cell cycle phase transition \| 219 \| 30 \| 13.83 \| 2.17 \| + \| 2.17E-04 \| 3.16E-02 \| \| regulation of mitotic cell cycle phase transition \| 419 \| 53 \| 26.47 \| 2.00 \| + \| 7.69E-06 \| 2.03E-03 \| \| regulation of cell cycle phase transition \| 457 \| 56 \| 28.87 \| 1.94 \| + \| 1.10E-05 \| 2.76E-03 \| \| regulation of cell cycle process \| 769 \| 85 \| 48.57 \| 1.75 \| + \| 2.79E-06 \| 7.90E-04 \| \| negative regulation of cell cycle process \| 329 \| 41 \| 20.78 \| 1.97 \| + \| 1.31E-04 \| 2.21E-02 \| \| macromolecule methylation \| 261 \| 35 \| 16.49 \| 2.12 \| + \| 9.85E-05 \| 1.80E-02 \| \| macromolecule modification \| 3412 \| 275 \| 215.51 \| 1.28 \| + \| 3.14E-05 \| 6.63E-03 \| \| cellular macromolecule metabolic process \| 5145 \| 399 \| 324.97 \| 1.23 \| + \| 7.91E-06 \| 2.06E-03 \| \| methylation \| 319 \| 39 \| 20.15 \| 1.94 \| + \| 2.61E-04 \| 3.63E-02 \| \| microtubule cytoskeleton organization \| 578 \| 61 \| 36.51 \| 1.67 \| + \| 2.43E-04 \| 3.41E-02 \| \| cytoskeleton organization \| 1224 \| 118 \| 77.31 \| 1.53 \| + \| 1.65E-05 \| 3.90E-03 \| \| apoptotic process \| 916 \| 89 \| 57.86 \| 1.54 \| + \| 1.61E-04 \| 2.50E-02 \| \| programmed cell death \| 1049 \| 99 \| 66.26 \| 1.49 \| + \| 1.88E-04 \| 2.79E-02 \| \| cell death \| 1086 \| 104 \| 68.59 \| 1.52 \| + \| 6.75E-05 \| 1.32E-02 \| \| negative regulation of cellular macromolecule biosynthetic process \| 1471 \| 134 \| 92.91 \| 1.44 \| + \| 5.91E-05 \| 1.19E-02 \| \| regulation of cellular macromolecule biosynthetic process \| 3912 \| 328 \| 247.09 \| 1.33 \| + \| 1.02E-07 \| 5.41E-05 \| \| regulation of macromolecule biosynthetic process \| 4013 \| 335 \| 253.47 \| 1.32 \| + \| 1.11E-07 \| 5.49E-05 \| \| regulation of biosynthetic process \| 4253 \| 346 \| 268.63 \| 1.29 \| + \| 7.42E-07 \| 2.74E-04 \| \| regulation of cellular biosynthetic process \| 4172 \| 340 \| 263.51 \| 1.29 \| + \| 7.51E-07 \| 2.71E-04 \| \| negative regulation of macromolecule biosynthetic process \| 1522 \| 136 \| 96.13 \| 1.41 \| + \| 1.20E-04 \| 2.04E-02 \| \| negative regulation of biosynthetic process \| 1613 \| 142 \| 101.88 \| 1.39 \| + \| 1.45E-04 \| 2.30E-02 \| \| negative regulation of cellular biosynthetic process \| 1584 \| 141 \| 100.05 \| 1.41 \| + \| 1.03E-04 \| 1.81E-02 \| \| negative regulation of cellular metabolic process \| 2654 \| 214 \| 167.63 \| 1.28 \| + \| 3.17E-04 \| 4.37E-02 \| \| regulation of multicellular organismal development \| 1332 \| 119 \| 84.13 \| 1.41 \| + \| 3.53E-04 \| 4.79E-02 \| \| regulation of developmental process \| 2469 \| 204 \| 155.95 \| 1.31 \| + \| 1.43E-04 \| 2.29E-02 \| \| regulation of cellular component organization \| 2373 \| 197 \| 149.88 \| 1.31 \| + \| 1.33E-04 \| 2.22E-02 \| \| regulation of transcription by RNA polymerase II \| 2629 \| 215 \| 166.05 \| 1.29 \| + \| 1.52E-04 \| 2.39E-02 \| \| regulation of transcription, DNA-templated \| 3452 \| 283 \| 218.04 \| 1.30 \| + \| 7.20E-06 \| 1.94E-03 \| \| regulation of nucleic acid-templated transcription \| 3521 \| 286 \| 222.39 \| 1.29 \| + \| 1.20E-05 \| 2.98E-03 \| \| regulation of RNA biosynthetic process \| 3526 \| 286 \| 222.71 \| 1.28 \| + \| 1.44E-05 \| 3.46E-03 \| \| regulation of cellular protein metabolic process \| 2668 \| 218 \| 168.52 \| 1.29 \| + \| 1.41E-04 \| 2.31E-02 \| \| regulation of protein metabolic process \| 2806 \| 229 \| 177.23 \| 1.29 \| + \| 9.95E-05 \| 1.79E-02 \| \| positive regulation of cellular metabolic process \| 3392 \| 275 \| 214.25 \| 1.28 \| + \| 2.13E-05 \| 4.77E-03 \| \| positive regulation of cellular process \| 5695 \| 434 \| 359.71 \| 1.21 \| + \| 1.32E-05 \| 3.22E-03 \| \| positive regulation of biological process \| 6290 \| 466 \| 397.29 \| 1.17 \| + \| 8.73E-05 \| 1.67E-02 \| \| positive regulation of metabolic process \| 3861 \| 302 \| 243.87 \| 1.24 \| + \| 9.80E-05 \| 1.81E-02 \| \| positive regulation of nitrogen compound metabolic process \| 3206 \| 255 \| 202.50 \| 1.26 \| + \| 1.68E-04 \| 2.59E-02 \| \| positive regulation of macromolecule metabolic process \| 3571 \| 279 \| 225.55 \| 1.24 \| + \| 2.34E-04 \| 3.37E-02 \| \| Unclassified \| 2839 \| 108 \| 179.32 \| .60 \| - \| 3.20E-09 \| 3.17E-06 \| \| humoral immune response \| 386 \| 7 \| 24.38 \| .29 \| - \| 8.86E-05 \| 1.67E-02 \| \| adaptive immune response \| 628 \| 11 \| 39.67 \| .28 \| - \| 2.39E-07 \| 1.05E-04 \| \| detection of chemical stimulus involved in sensory perception of smell \| 440 \| 0 \| 27.79 \| < 0.01 \| - \| 2.64E-12 \| 4.19E-08 \| \| detection of chemical stimulus involved in sensory perception \| 485 \| 1 \| 30.63 \| .03 \| - \| 4.57E-12 \| 3.63E-08 \| \| detection of stimulus involved in sensory perception \| 550 \| 4 \| 34.74 \| .12 \| - \| 2.17E-10 \| 3.83E-07 \| \| sensory perception \| 981 \| 29 \| 61.96 \| .47 \| - \| 5.97E-06 \| 1.63E-03 \| \| nervous system process \| 1403 \| 56 \| 88.62 \| .63 \| - \| 2.39E-04 \| 3.39E-02 \| \| detection of stimulus \| 712 \| 8 \| 44.97 \| .18 \| - \| 3.82E-11 \| 2.02E-07 \| \| detection of chemical stimulus \| 521 \| 3 \| 32.91 \| .09 \| - \| 1.19E-10 \| 3.16E-07 \| \| sensory perception of chemical stimulus \| 538 \| 7 \| 33.98 \| .21 \| - \| 6.28E-08 \| 3.44E-05 \| \| sensory perception of smell \| 466 \| 3 \| 29.43 \| .10 \| - \| 2.35E-09 \| 2.86E-06 \| |
| --- | --- | --- | --- | --- | --- | --- | --- | --- | --- | --- | --- | --- | --- | --- | --- | --- | --- | --- | --- | --- | --- | --- | --- | --- | --- | --- | --- | --- | --- | --- | --- | --- | --- | --- | --- | --- | --- | --- | --- | --- | --- | --- | --- | --- | --- | --- | --- | --- | --- | --- | --- | --- | --- | --- | --- | --- | --- | --- | --- | --- | --- | --- | --- | --- | --- | --- | --- | --- | --- | --- | --- | --- | --- | --- | --- | --- | --- | --- | --- | --- | --- | --- | --- | --- | --- | --- | --- | --- | --- | --- | --- | --- | --- | --- | --- | --- | --- | --- | --- | --- | --- | --- | --- | --- | --- | --- | --- | --- | --- | --- | --- | --- | --- | --- | --- | --- | --- | --- | --- | --- | --- | --- | --- | --- | --- | --- | --- | --- | --- | --- | --- | --- | --- | --- | --- | --- | --- | --- | --- | --- | --- | --- | --- | --- | --- | --- | --- | --- | --- | --- | --- | --- | --- | --- | --- | --- | --- | --- | --- | --- | --- | --- | --- | --- | --- | --- | --- | --- | --- | --- | --- | --- | --- | --- | --- | --- | --- | --- | --- | --- | --- | --- | --- | --- | --- | --- | --- | --- | --- | --- | --- | --- | --- | --- | --- | --- | --- | --- | --- | --- | --- | --- | --- | --- | --- | --- | --- | --- | --- | --- | --- | --- | --- | --- | --- | --- | --- | --- | --- | --- | --- | --- | --- | --- | --- | --- | --- | --- | --- | --- | --- | --- | --- | --- | --- | --- | --- | --- | --- | --- | --- | --- | --- | --- | --- | --- | --- | --- | --- | --- | --- | --- | --- | --- | --- | --- | --- | --- | --- | --- | --- | --- | --- | --- | --- | --- | --- | --- | --- | --- | --- | --- | --- | --- | --- | --- | --- | --- | --- | --- | --- | --- | --- | --- | --- | --- | --- | --- | --- | --- | --- | --- | --- | --- | --- | --- | --- | --- | --- | --- | --- | --- | --- | --- | --- | --- | --- | --- | --- | --- | --- | --- | --- | --- | --- | --- | --- | --- | --- | --- | --- | --- | --- | --- | --- | --- | --- | --- | --- | --- | --- | --- | --- | --- | --- | --- | --- | --- | --- | --- | --- | --- | --- | --- | --- | --- | --- | --- | --- | --- | --- | --- | --- | --- | --- | --- | --- | --- | --- | --- | --- | --- | --- | --- | --- | --- | --- | --- | --- | --- | --- | --- | --- | --- | --- | --- | --- | --- | --- | --- | --- | --- | --- | --- | --- | --- | --- | --- | --- | --- | --- | --- | --- | --- | --- | --- | --- | --- | --- | --- | --- | --- | --- | --- | --- | --- | --- | --- | --- | --- | --- | --- | --- | --- | --- | --- | --- | --- | --- | --- | --- | --- | --- | --- | --- | --- | --- | --- | --- | --- | --- | --- | --- | --- | --- | --- | --- | --- | --- | --- | --- | --- | --- | --- | --- | --- | --- | --- | --- | --- | --- | --- | --- | --- | --- | --- | --- | --- | --- | --- | --- | --- | --- | --- | --- | --- | --- | --- | --- | --- | --- | --- | --- | --- | --- | --- | --- | --- | --- | --- | --- | --- | --- | --- | --- | --- | --- | --- | --- | --- | --- | --- | --- | --- | --- | --- | --- | --- | --- | --- | --- | --- | --- | --- | --- | --- | --- | --- | --- | --- | --- | --- | --- | --- | --- | --- | --- | --- | --- | --- | --- | --- | --- | --- | --- | --- | --- | --- | --- | --- | --- | --- | --- | --- | --- | --- | --- | --- | --- | --- | --- | --- | --- | --- | --- | --- | --- | --- | --- | --- | --- | --- | --- | --- | --- | --- | --- | --- | --- | --- | --- | --- | --- | --- | --- | --- | --- | --- | --- | --- | --- | --- | --- | --- | --- | --- | --- | --- | --- | --- | --- | --- | --- | --- | --- | --- | --- | --- | --- | --- | --- | --- | --- | --- | --- | --- | --- | --- | --- | --- | --- | --- | --- | --- | --- | --- | --- | --- | --- | --- | --- | --- | --- | --- | --- | --- | --- | --- | --- | --- | --- | --- | --- | --- | --- | --- | --- | --- | --- | --- | --- | --- | --- | --- | --- | --- | --- | --- | --- | --- | --- | --- | --- | --- | --- | --- | --- | --- | --- | --- | --- | --- | --- | --- | --- | --- | --- | --- | --- | --- | --- | --- | --- | --- | --- | --- | --- | --- | --- | --- | --- | --- | --- | --- | --- | --- | --- | --- | --- | --- | --- | --- | --- | --- | --- | --- | --- | --- | --- | --- | --- | --- | --- | --- | --- | --- | --- | --- | --- | --- | --- | --- | --- | --- | --- | --- | --- | --- | --- | --- | --- | --- | --- | --- | --- | --- | --- | --- | --- | --- | --- | --- | --- | --- | --- | --- | --- | --- | --- | --- | --- | --- | --- | --- | --- | --- | --- | --- | --- | --- | --- | --- | --- | --- | --- | --- | --- | --- | --- | --- | --- | --- | --- | --- | --- | --- | --- | --- | --- | --- | --- | --- | --- | --- | --- | --- | --- | --- | --- | --- | --- | --- | --- | --- | --- | --- | --- | --- | --- | --- | --- | --- | --- | --- | --- | --- | --- | --- | --- | --- | --- | --- | --- | --- | --- | --- | --- | --- | --- | --- | --- | --- | --- | --- | --- | --- | --- | --- | --- | --- | --- | --- | --- | --- | --- | --- | --- | --- | --- | --- | --- | --- | --- | --- | --- | --- | --- | --- | --- | --- | --- | --- | --- | --- | --- | --- | --- | --- | --- | --- | --- | --- | --- | --- | --- | --- | --- | --- | --- | --- | --- | --- | --- | --- | --- | --- | --- | --- | --- | --- | --- | --- | --- | --- | --- | --- | --- | --- | --- | --- | --- | --- | --- | --- | --- | --- | --- | --- | --- | --- | --- | --- | --- | --- | --- | --- | --- | --- | --- | --- | --- | --- | --- | --- | --- | --- | --- | --- | --- | --- | --- | --- | --- | --- | --- | --- | --- | --- | --- | --- | --- | --- | --- | --- | --- | --- | --- | --- | --- | --- | --- | --- | --- | --- | --- | --- | --- | --- | --- | --- | --- | --- | --- | --- | --- | --- | --- | --- | --- | --- | --- | --- | --- | --- | --- | --- | --- | --- | --- | --- | --- | --- |

S8. MOLECULAR FUNCTIONS

|  | Homo sapiens | RESULTS | | | | | |
| --- | --- | --- | --- | --- | --- | --- | --- |
| GO molecular function complete | # | # | expected | Fold Enrichment | +/- | raw P value | FDR |
| mRNA binding | 301 | 43 | 19.01 | 2.26 | + | 4.53E-06 | 2.41E-03 |
| RNA binding | 1691 | 152 | 106.81 | 1.42 | + | 2.91E-05 | 1.16E-02 |
| nucleic acid binding | 4007 | 341 | 253.09 | 1.35 | + | 9.62E-09 | 5.75E-06 |
| organic cyclic compound binding | 6066 | 504 | 383.14 | 1.32 | + | 5.32E-12 | 4.24E-09 |
| binding | 16634 | 1183 | 1050.64 | 1.13 | + | 1.76E-21 | 8.42E-18 |
| heterocyclic compound binding | 5977 | 497 | 377.52 | 1.32 | + | 6.78E-12 | 4.63E-09 |
| DNA binding | 2499 | 212 | 157.84 | 1.34 | + | 2.06E-05 | 8.93E-03 |
| enzyme binding | 2302 | 192 | 145.40 | 1.32 | + | 1.30E-04 | 3.89E-02 |
| protein binding | 14393 | 1052 | 909.10 | 1.16 | + | 8.05E-18 | 1.92E-14 |
| metal ion binding | 4289 | 335 | 270.90 | 1.24 | + | 3.63E-05 | 1.24E-02 |
| cation binding | 4376 | 338 | 276.40 | 1.22 | + | 8.56E-05 | 2.73E-02 |
| ion binding | 6397 | 484 | 404.05 | 1.20 | + | 5.37E-06 | 2.57E-03 |
| G protein-coupled receptor activity | 884 | 27 | 55.84 | .48 | - | 3.03E-05 | 1.11E-02 |
| Unclassified | 2468 | 69 | 155.88 | .44 | - | 1.67E-15 | 1.99E-12 |
| olfactory receptor activity | 440 | 0 | 27.79 | < 0.01 | - | 2.64E-12 | 2.53E-09 |

S9. CELLULAR COMPONENTS

|  | Homo sapiens | RESULTS | | | | | |
| --- | --- | --- | --- | --- | --- | --- | --- |
| GO cellular component complete | # | # | expected | Fold Enrichment | +/- | raw P value | FDR |
| ciliary tip | 47 | 13 | 2.97 | 4.38 | + | 4.07E-05 | 2.92E-03 |
| cellular anatomical entity | 18825 | 1260 | 1189.03 | 1.06 | + | 1.16E-12 | 2.58E-10 |
| cilium | 683 | 81 | 43.14 | 1.88 | + | 4.61E-07 | 3.85E-05 |
| organelle | 13908 | 1056 | 878.46 | 1.20 | + | 5.91E-26 | 3.95E-23 |
| plasma membrane bounded cell projection | 2256 | 189 | 142.49 | 1.33 | + | 1.35E-04 | 8.72E-03 |
| cell projection | 2354 | 201 | 148.68 | 1.35 | + | 2.31E-05 | 1.71E-03 |
| ciliary basal body | 157 | 32 | 9.92 | 3.23 | + | 7.64E-08 | 6.66E-06 |
| microtubule organizing center | 799 | 102 | 50.47 | 2.02 | + | 3.39E-10 | 3.77E-08 |
| microtubule cytoskeleton | 1285 | 138 | 81.16 | 1.70 | + | 7.70E-09 | 7.35E-07 |
| cytoskeleton | 2311 | 207 | 145.97 | 1.42 | + | 9.75E-07 | 7.82E-05 |
| intracellular non-membrane-bounded organelle | 5304 | 444 | 335.01 | 1.33 | + | 9.85E-11 | 1.23E-08 |
| intracellular organelle | 13102 | 1023 | 827.55 | 1.24 | + | 3.10E-29 | 3.11E-26 |
| intracellular anatomical structure | 14899 | 1142 | 941.06 | 1.21 | + | 1.24E-37 | 2.48E-34 |
| non-membrane-bounded organelle | 5307 | 444 | 335.20 | 1.32 | + | 1.00E-10 | 1.18E-08 |
| centriolar satellite | 97 | 19 | 6.13 | 3.10 | + | 5.19E-05 | 3.59E-03 |
| centrosome | 602 | 80 | 38.02 | 2.10 | + | 5.12E-09 | 5.14E-07 |
| centriole | 148 | 24 | 9.35 | 2.57 | + | 1.11E-04 | 7.39E-03 |
| nuclear speck | 403 | 60 | 25.45 | 2.36 | + | 1.25E-08 | 1.14E-06 |
| nuclear body | 796 | 98 | 50.28 | 1.95 | + | 4.84E-09 | 5.11E-07 |
| nucleoplasm | 3991 | 364 | 252.08 | 1.44 | + | 5.23E-13 | 1.31E-10 |
| nuclear lumen | 4992 | 430 | 315.31 | 1.36 | + | 5.24E-12 | 1.05E-09 |
| intracellular organelle lumen | 6118 | 501 | 386.43 | 1.30 | + | 6.27E-11 | 8.99E-09 |
| organelle lumen | 6118 | 501 | 386.43 | 1.30 | + | 6.27E-11 | 9.68E-09 |
| membrane-enclosed lumen | 6118 | 501 | 386.43 | 1.30 | + | 6.27E-11 | 8.39E-09 |
| nucleus | 7603 | 645 | 480.22 | 1.34 | + | 2.84E-19 | 1.14E-16 |
| intracellular membrane-bounded organelle | 11304 | 885 | 713.99 | 1.24 | + | 1.48E-20 | 7.43E-18 |
| membrane-bounded organelle | 12791 | 965 | 807.91 | 1.19 | + | 1.11E-18 | 3.72E-16 |
| cytosol | 5305 | 415 | 335.08 | 1.24 | + | 1.75E-06 | 1.35E-04 |
| cytoplasm | 11958 | 904 | 755.30 | 1.20 | + | 3.70E-16 | 1.06E-13 |
| Unclassified | 1887 | 55 | 119.19 | .46 | - | 4.67E-11 | 8.51E-09 |
| immunoglobulin complex | 188 | 1 | 11.87 | .08 | - | 2.63E-04 | 1.65E-02 |
| protein-containing complex | 5563 | 409 | 351.37 | 1.16 | + | 6.08E-04 | 3.70E-02 |

1. **SHANNON ENTROPY (SE) CRITERION**

Considered set: Probes changing their Shannon Entropy from nulliparous to parous, adjusted *p*-value<0.05, 20% with higher relative change.

*B1. Higher SE in parous.*

S10. BIOLOGICAL PROCESSES

| \|  \| Homo sapiens \| RESULTS \| \| \| \| \| \| \| --- \| --- \| --- \| --- \| --- \| --- \| --- \| --- \| \| GO biological process complete \| **#** \| **#** \| expected \| Fold Enrichment \| +/- \| raw P value \| FDR \| \| \| valyl-tRNA aminoacylation \| 6 \| 6 \| .18 \| 33.20 \| + \| 4.85E-07 \| 2.08E-04 \| \| \| RNA metabolic process \| 1619 \| 95 \| 48.76 \| 1.95 \| + \| 1.30E-09 \| 1.37E-06 \| \| \| nucleic acid metabolic process \| 2241 \| 123 \| 67.50 \| 1.82 \| + \| 1.43E-10 \| 2.06E-07 \| \| \| nucleobase-containing compound metabolic process \| 2743 \| 139 \| 82.61 \| 1.68 \| + \| 1.40E-09 \| 1.38E-06 \| \| \| heterocycle metabolic process \| 2937 \| 146 \| 88.46 \| 1.65 \| + \| 1.43E-09 \| 1.34E-06 \| \| \| cellular metabolic process \| 7784 \| 331 \| 234.44 \| 1.41 \| + \| 1.38E-14 \| 2.20E-10 \| \| \| metabolic process \| 8592 \| 350 \| 258.78 \| 1.35 \| + \| 6.30E-13 \| 1.67E-09 \| \| \| cellular process \| 15598 \| 529 \| 469.79 \| 1.13 \| + \| 2.17E-08 \| 1.33E-05 \| \| \| primary metabolic process \| 7578 \| 322 \| 228.24 \| 1.41 \| + \| 7.01E-14 \| 3.71E-10 \| \| \| cellular aromatic compound metabolic process \| 2987 \| 150 \| 89.96 \| 1.67 \| + \| 4.17E-10 \| 5.51E-07 \| \| \| cellular nitrogen compound metabolic process \| 3405 \| 161 \| 102.55 \| 1.57 \| + \| 6.21E-09 \| 3.94E-06 \| \| \| nitrogen compound metabolic process \| 7091 \| 305 \| 213.57 \| 1.43 \| + \| 1.55E-13 \| 6.15E-10 \| \| \| organic cyclic compound metabolic process \| 3220 \| 163 \| 96.98 \| 1.68 \| + \| 2.50E-11 \| 5.67E-08 \| \| \| organic substance metabolic process \| 8011 \| 336 \| 241.28 \| 1.39 \| + \| 5.14E-14 \| 4.08E-10 \| \| \| macromolecule metabolic process \| 6333 \| 279 \| 190.74 \| 1.46 \| + \| 3.10E-13 \| 9.84E-10 \| \| \| organonitrogen compound metabolic process \| 5444 \| 213 \| 163.96 \| 1.30 \| + \| 2.22E-05 \| 5.18E-03 \| \| \| gene expression \| 2105 \| 113 \| 63.40 \| 1.78 \| + \| 3.34E-09 \| 2.31E-06 \| \| \| cellular macromolecule metabolic process \| 5145 \| 218 \| 154.96 \| 1.41 \| + \| 2.99E-08 \| 1.69E-05 \| \| \| cellular protein metabolic process \| 3822 \| 160 \| 115.11 \| 1.39 \| + \| 1.42E-05 \| 3.74E-03 \| \| \| protein metabolic process \| 4407 \| 181 \| 132.73 \| 1.36 \| + \| 8.12E-06 \| 2.38E-03 \| \| \| aminoacyl-tRNA metabolism involved in translational fidelity \| 18 \| 7 \| .54 \| 12.91 \| + \| 5.37E-06 \| 1.67E-03 \| \| \| regulation of translational fidelity \| 26 \| 7 \| .78 \| 8.94 \| + \| 3.89E-05 \| 8.70E-03 \| \| \| biological regulation \| 12645 \| 437 \| 380.85 \| 1.15 \| + \| 4.77E-06 \| 1.51E-03 \| \| \| intraciliary transport involved in cilium assembly \| 40 \| 8 \| 1.20 \| 6.64 \| + \| 6.86E-05 \| 1.47E-02 \| \| \| cilium assembly \| 359 \| 27 \| 10.81 \| 2.50 \| + \| 3.90E-05 \| 8.59E-03 \| \| \| organelle assembly \| 773 \| 50 \| 23.28 \| 2.15 \| + \| 1.26E-06 \| 4.65E-04 \| \| \| organelle organization \| 3586 \| 146 \| 108.00 \| 1.35 \| + \| 1.42E-04 \| 2.85E-02 \| \| \| cellular component organization \| 5703 \| 224 \| 171.77 \| 1.30 \| + \| 8.47E-06 \| 2.44E-03 \| \| \| cellular component organization or biogenesis \| 5922 \| 233 \| 178.36 \| 1.31 \| + \| 3.71E-06 \| 1.25E-03 \| \| \| cellular component assembly \| 2394 \| 111 \| 72.10 \| 1.54 \| + \| 6.72E-06 \| 2.01E-03 \| \| \| cellular component biogenesis \| 2654 \| 122 \| 79.93 \| 1.53 \| + \| 3.00E-06 \| 1.06E-03 \| \| \| plasma membrane bounded cell projection assembly \| 453 \| 30 \| 13.64 \| 2.20 \| + \| 1.61E-04 \| 3.20E-02 \| \| \| cell projection assembly \| 470 \| 30 \| 14.16 \| 2.12 \| + \| 2.19E-04 \| 4.05E-02 \| \| \| cilium organization \| 382 \| 29 \| 11.51 \| 2.52 \| + \| 1.73E-05 \| 4.22E-03 \| \| \| intraciliary transport \| 53 \| 10 \| 1.60 \| 6.26 \| + \| 1.34E-05 \| 3.61E-03 \| \| \| protein-containing complex localization \| 255 \| 20 \| 7.68 \| 2.60 \| + \| 1.85E-04 \| 3.54E-02 \| \| \| protein transport along microtubule \| 66 \| 11 \| 1.99 \| 5.53 \| + \| 1.42E-05 \| 3.69E-03 \| \| \| microtubule-based protein transport \| 66 \| 11 \| 1.99 \| 5.53 \| + \| 1.42E-05 \| 3.63E-03 \| \| \| non-motile cilium assembly \| 49 \| 9 \| 1.48 \| 6.10 \| + \| 4.37E-05 \| 9.50E-03 \| \| \| spliceosomal complex assembly \| 56 \| 9 \| 1.69 \| 5.34 \| + \| 1.09E-04 \| 2.25E-02 \| \| \| mRNA splicing, via spliceosome \| 304 \| 28 \| 9.16 \| 3.06 \| + \| 5.90E-07 \| 2.46E-04 \| \| \| mRNA processing \| 489 \| 40 \| 14.73 \| 2.72 \| + \| 4.76E-08 \| 2.52E-05 \| \| \| RNA processing \| 929 \| 65 \| 27.98 \| 2.32 \| + \| 1.54E-09 \| 1.36E-06 \| \| \| mRNA metabolic process \| 700 \| 48 \| 21.08 \| 2.28 \| + \| 3.73E-07 \| 1.64E-04 \| \| \| RNA splicing, via transesterification reactions with bulged adenosine as nucleophile \| 304 \| 28 \| 9.16 \| 3.06 \| + \| 5.90E-07 \| 2.40E-04 \| \| \| RNA splicing, via transesterification reactions \| 307 \| 28 \| 9.25 \| 3.03 \| + \| 7.07E-07 \| 2.80E-04 \| \| \| RNA splicing \| 410 \| 39 \| 12.35 \| 3.16 \| + \| 1.57E-09 \| 1.31E-06 \| \| \| regulation of RNA splicing \| 158 \| 17 \| 4.76 \| 3.57 \| + \| 1.58E-05 \| 3.98E-03 \| \| \| regulation of gene expression \| 4894 \| 216 \| 147.40 \| 1.47 \| + \| 1.23E-09 \| 1.40E-06 \| \| \| regulation of macromolecule metabolic process \| 6491 \| 258 \| 195.50 \| 1.32 \| + \| 2.39E-07 \| 1.08E-04 \| \| \| regulation of metabolic process \| 7025 \| 279 \| 211.58 \| 1.32 \| + \| 4.01E-08 \| 2.20E-05 \| \| \| regulation of biological process \| 11960 \| 406 \| 360.22 \| 1.13 \| + \| 2.61E-04 \| 4.76E-02 \| \| \| regulation of RNA metabolic process \| 3801 \| 182 \| 114.48 \| 1.59 \| + \| 1.06E-10 \| 1.68E-07 \| \| \| regulation of nucleobase-containing compound metabolic process \| 4073 \| 189 \| 122.67 \| 1.54 \| + \| 6.08E-10 \| 7.42E-07 \| \| \| regulation of nitrogen compound metabolic process \| 5895 \| 236 \| 177.55 \| 1.33 \| + \| 7.45E-07 \| 2.88E-04 \| \| \| regulation of cellular metabolic process \| 6304 \| 257 \| 189.87 \| 1.35 \| + \| 2.64E-08 \| 1.55E-05 \| \| \| regulation of primary metabolic process \| 6086 \| 246 \| 183.30 \| 1.34 \| + \| 1.43E-07 \| 6.90E-05 \| \| \| gene silencing \| 152 \| 15 \| 4.58 \| 3.28 \| + \| 1.20E-04 \| 2.45E-02 \| \| \| negative regulation of gene expression \| 2024 \| 97 \| 60.96 \| 1.59 \| + \| 8.93E-06 \| 2.53E-03 \| \| \| negative regulation of macromolecule metabolic process \| 2908 \| 122 \| 87.58 \| 1.39 \| + \| 1.89E-04 \| 3.56E-02 \| \| \| negative regulation of metabolic process \| 3155 \| 132 \| 95.02 \| 1.39 \| + \| 1.00E-04 \| 2.09E-02 \| \| \| negative regulation of cellular macromolecule biosynthetic process \| 1471 \| 76 \| 44.30 \| 1.72 \| + \| 9.56E-06 \| 2.61E-03 \| \| \| regulation of cellular macromolecule biosynthetic process \| 3912 \| 181 \| 117.82 \| 1.54 \| + \| 1.77E-09 \| 1.41E-06 \| \| \| regulation of macromolecule biosynthetic process \| 4013 \| 184 \| 120.87 \| 1.52 \| + \| 2.51E-09 \| 1.81E-06 \| \| \| regulation of biosynthetic process \| 4253 \| 193 \| 128.09 \| 1.51 \| + \| 2.14E-09 \| 1.62E-06 \| \| \| regulation of cellular biosynthetic process \| 4172 \| 189 \| 125.65 \| 1.50 \| + \| 4.17E-09 \| 2.76E-06 \| \| \| negative regulation of macromolecule biosynthetic process \| 1522 \| 78 \| 45.84 \| 1.70 \| + \| 9.19E-06 \| 2.56E-03 \| \| \| negative regulation of biosynthetic process \| 1613 \| 82 \| 48.58 \| 1.69 \| + \| 5.70E-06 \| 1.74E-03 \| \| \| negative regulation of cellular biosynthetic process \| 1584 \| 82 \| 47.71 \| 1.72 \| + \| 3.18E-06 \| 1.10E-03 \| \| \| negative regulation of cellular metabolic process \| 2654 \| 113 \| 79.93 \| 1.41 \| + \| 2.15E-04 \| 4.01E-02 \| \| \| regulation of transcription by RNA polymerase II \| 2629 \| 116 \| 79.18 \| 1.46 \| + \| 3.49E-05 \| 7.92E-03 \| \| \| regulation of transcription, DNA-templated \| 3452 \| 157 \| 103.97 \| 1.51 \| + \| 1.26E-07 \| 6.25E-05 \| \| \| regulation of nucleic acid-templated transcription \| 3521 \| 159 \| 106.05 \| 1.50 \| + \| 1.58E-07 \| 7.38E-05 \| \| \| regulation of RNA biosynthetic process \| 3526 \| 160 \| 106.20 \| 1.51 \| + \| 9.12E-08 \| 4.67E-05 \| \| \| macromolecule modification \| 3412 \| 145 \| 102.76 \| 1.41 \| + \| 1.99E-05 \| 4.78E-03 \| \| \| Unclassified \| 2839 \| 34 \| 85.51 \| .40 \| - \| 5.71E-11 \| 1.13E-07 \| \| \| adaptive immune response \| 628 \| 4 \| 18.91 \| .21 \| - \| 9.50E-05 \| 2.01E-02 \| \| \| detection of chemical stimulus involved in sensory perception of smell \| 440 \| 0 \| 13.25 \| < 0.01 \| - \| 4.30E-06 \| 1.42E-03 \| \| \| detection of chemical stimulus involved in sensory perception \| 485 \| 0 \| 14.61 \| < 0.01 \| - \| 8.28E-07 \| 3.13E-04 \| \| \| detection of stimulus involved in sensory perception \| 550 \| 1 \| 16.57 \| .06 \| - \| 2.18E-06 \| 7.87E-04 \| \| \| sensory perception \| 981 \| 9 \| 29.55 \| .30 \| - \| 1.71E-05 \| 4.24E-03 \| \| \| nervous system process \| 1403 \| 20 \| 42.26 \| .47 \| - \| 1.65E-04 \| 3.24E-02 \| \| \| detection of stimulus \| 712 \| 6 \| 21.44 \| .28 \| - \| 1.68E-04 \| 3.25E-02 \| \| \| detection of chemical stimulus \| 521 \| 1 \| 15.69 \| .06 \| - \| 4.63E-06 \| 1.50E-03 \| \| \| sensory perception of chemical stimulus \| 538 \| 2 \| 16.20 \| .12 \| - \| 2.47E-05 \| 5.67E-03 \| \| \| sensory perception of smell \| 466 \| 1 \| 14.04 \| .07 \| - \| 2.10E-05 \| 4.98E-03 \| \| |
| --- | --- | --- | --- | --- | --- | --- | --- | --- | --- | --- | --- | --- | --- | --- | --- | --- | --- | --- | --- | --- | --- | --- | --- | --- | --- | --- | --- | --- | --- | --- | --- | --- | --- | --- | --- | --- | --- | --- | --- | --- | --- | --- | --- | --- | --- | --- | --- | --- | --- | --- | --- | --- | --- | --- | --- | --- | --- | --- | --- | --- | --- | --- | --- | --- | --- | --- | --- | --- | --- | --- | --- | --- | --- | --- | --- | --- | --- | --- | --- | --- | --- | --- | --- | --- | --- | --- | --- | --- | --- | --- | --- | --- | --- | --- | --- | --- | --- | --- | --- | --- | --- | --- | --- | --- | --- | --- | --- | --- | --- | --- | --- | --- | --- | --- | --- | --- | --- | --- | --- | --- | --- | --- | --- | --- | --- | --- | --- | --- | --- | --- | --- | --- | --- | --- | --- | --- | --- | --- | --- | --- | --- | --- | --- | --- | --- | --- | --- | --- | --- | --- | --- | --- | --- | --- | --- | --- | --- | --- | --- | --- | --- | --- | --- | --- | --- | --- | --- | --- | --- | --- | --- | --- | --- | --- | --- | --- | --- | --- | --- | --- | --- | --- | --- | --- | --- | --- | --- | --- | --- | --- | --- | --- | --- | --- | --- | --- | --- | --- | --- | --- | --- | --- | --- | --- | --- | --- | --- | --- | --- | --- | --- | --- | --- | --- | --- | --- | --- | --- | --- | --- | --- | --- | --- | --- | --- | --- | --- | --- | --- | --- | --- | --- | --- | --- | --- | --- | --- | --- | --- | --- | --- | --- | --- | --- | --- | --- | --- | --- | --- | --- | --- | --- | --- | --- | --- | --- | --- | --- | --- | --- | --- | --- | --- | --- | --- | --- | --- | --- | --- | --- | --- | --- | --- | --- | --- | --- | --- | --- | --- | --- | --- | --- | --- | --- | --- | --- | --- | --- | --- | --- | --- | --- | --- | --- | --- | --- | --- | --- | --- | --- | --- | --- | --- | --- | --- | --- | --- | --- | --- | --- | --- | --- | --- | --- | --- | --- | --- | --- | --- | --- | --- | --- | --- | --- | --- | --- | --- | --- | --- | --- | --- | --- | --- | --- | --- | --- | --- | --- | --- | --- | --- | --- | --- | --- | --- | --- | --- | --- | --- | --- | --- | --- | --- | --- | --- | --- | --- | --- | --- | --- | --- | --- | --- | --- | --- | --- | --- | --- | --- | --- | --- | --- | --- | --- | --- | --- | --- | --- | --- | --- | --- | --- | --- | --- | --- | --- | --- | --- | --- | --- | --- | --- | --- | --- | --- | --- | --- | --- | --- | --- | --- | --- | --- | --- | --- | --- | --- | --- | --- | --- | --- | --- | --- | --- | --- | --- | --- | --- | --- | --- | --- | --- | --- | --- | --- | --- | --- | --- | --- | --- | --- | --- | --- | --- | --- | --- | --- | --- | --- | --- | --- | --- | --- | --- | --- | --- | --- | --- | --- | --- | --- | --- | --- | --- | --- | --- | --- | --- | --- | --- | --- | --- | --- | --- | --- | --- | --- | --- | --- | --- | --- | --- | --- | --- | --- | --- | --- | --- | --- | --- | --- | --- | --- | --- | --- | --- | --- | --- | --- | --- | --- | --- | --- | --- | --- | --- | --- | --- | --- | --- | --- | --- | --- | --- | --- | --- | --- | --- | --- | --- | --- | --- | --- | --- | --- | --- | --- | --- | --- | --- | --- | --- | --- | --- | --- | --- | --- | --- | --- | --- | --- | --- | --- | --- | --- | --- | --- | --- | --- | --- | --- | --- | --- | --- | --- | --- | --- | --- | --- | --- | --- | --- | --- | --- | --- | --- | --- | --- | --- | --- | --- | --- | --- | --- | --- | --- | --- | --- | --- | --- | --- | --- | --- | --- | --- | --- | --- | --- | --- | --- | --- | --- | --- | --- | --- | --- | --- | --- | --- | --- | --- | --- | --- | --- | --- | --- | --- | --- | --- | --- | --- | --- | --- | --- | --- | --- | --- | --- | --- | --- | --- | --- | --- | --- | --- | --- | --- | --- | --- | --- | --- | --- | --- | --- | --- | --- | --- | --- | --- | --- | --- | --- | --- | --- | --- | --- | --- | --- | --- | --- | --- | --- | --- | --- | --- | --- | --- | --- | --- | --- | --- | --- | --- | --- | --- | --- | --- | --- | --- | --- | --- | --- | --- | --- | --- | --- | --- | --- | --- | --- | --- | --- | --- | --- | --- | --- | --- | --- | --- | --- | --- | --- | --- | --- | --- | --- | --- | --- | --- | --- | --- | --- | --- | --- | --- | --- | --- | --- | --- | --- | --- | --- | --- | --- | --- | --- | --- | --- | --- | --- | --- | --- | --- | --- | --- | --- | --- | --- | --- | --- | --- | --- | --- | --- | --- | --- | --- | --- | --- | --- | --- | --- | --- | --- | --- | --- | --- | --- | --- | --- | --- | --- | --- | --- | --- | --- | --- | --- | --- | --- | --- | --- | --- | --- | --- | --- | --- | --- | --- | --- | --- | --- | --- | --- | --- | --- | --- | --- | --- | --- | --- | --- | --- | --- | --- | --- | --- | --- | --- | --- | --- | --- | --- | --- | --- | --- | --- | --- | --- | --- | --- |

S11. MOLECULAR FUNCTIONS

|  | Homo sapiens | RESULTS | | | | | |
| --- | --- | --- | --- | --- | --- | --- | --- |
| GO molecular function complete | # | # | expected | Fold Enrichment | +/- | raw P value | FDR |
| valine-tRNA ligase activity | 6 | 6 | .18 | 33.20 | + | 4.85E-07 | 1.93E-04 |
| catalytic activity, acting on RNA | 409 | 27 | 12.32 | 2.19 | + | 2.55E-04 | 3.30E-02 |
| catalytic activity | 5840 | 239 | 175.89 | 1.36 | + | 9.98E-08 | 4.77E-05 |
| aminoacyl-tRNA editing activity | 18 | 7 | .54 | 12.91 | + | 5.37E-06 | 1.51E-03 |
| protein threonine phosphatase activity | 80 | 10 | 2.41 | 4.15 | + | 2.97E-04 | 3.74E-02 |
| catalytic activity, acting on a protein | 2271 | 104 | 68.40 | 1.52 | + | 2.46E-05 | 4.52E-03 |
| protein serine phosphatase activity | 80 | 10 | 2.41 | 4.15 | + | 2.97E-04 | 3.64E-02 |
| mRNA binding | 301 | 27 | 9.07 | 2.98 | + | 1.49E-06 | 4.76E-04 |
| RNA binding | 1691 | 88 | 50.93 | 1.73 | + | 1.00E-06 | 3.42E-04 |
| nucleic acid binding | 4007 | 190 | 120.68 | 1.57 | + | 8.76E-11 | 5.98E-08 |
| organic cyclic compound binding | 6066 | 277 | 182.70 | 1.52 | + | 4.62E-15 | 1.10E-11 |
| binding | 16634 | 574 | 500.99 | 1.15 | + | 4.38E-15 | 2.09E-11 |
| heterocyclic compound binding | 5977 | 271 | 180.02 | 1.51 | + | 3.03E-14 | 4.83E-11 |
| ATP binding | 1511 | 75 | 45.51 | 1.65 | + | 3.58E-05 | 6.12E-03 |
| adenyl ribonucleotide binding | 1574 | 79 | 47.41 | 1.67 | + | 1.33E-05 | 3.03E-03 |
| adenyl nucleotide binding | 1586 | 79 | 47.77 | 1.65 | + | 1.94E-05 | 4.02E-03 |
| purine nucleotide binding | 1943 | 90 | 58.52 | 1.54 | + | 7.31E-05 | 1.13E-02 |
| nucleotide binding | 2184 | 101 | 65.78 | 1.54 | + | 2.37E-05 | 4.71E-03 |
| small molecule binding | 2595 | 115 | 78.16 | 1.47 | + | 3.16E-05 | 5.59E-03 |
| nucleoside phosphate binding | 2185 | 101 | 65.81 | 1.53 | + | 2.38E-05 | 4.55E-03 |
| purine ribonucleotide binding | 1929 | 90 | 58.10 | 1.55 | + | 5.20E-05 | 8.28E-03 |
| ribonucleotide binding | 1946 | 90 | 58.61 | 1.54 | + | 7.42E-05 | 1.11E-02 |
| carbohydrate derivative binding | 2290 | 99 | 68.97 | 1.44 | + | 3.75E-04 | 4.48E-02 |
| purine ribonucleoside triphosphate binding | 1859 | 86 | 55.99 | 1.54 | + | 9.78E-05 | 1.42E-02 |
| anion binding | 2896 | 122 | 87.22 | 1.40 | + | 1.82E-04 | 2.48E-02 |
| ion binding | 6397 | 278 | 192.67 | 1.44 | + | 1.86E-12 | 1.48E-09 |
| double-stranded DNA binding | 1696 | 80 | 51.08 | 1.57 | + | 1.19E-04 | 1.68E-02 |
| DNA binding | 2499 | 114 | 75.27 | 1.51 | + | 9.96E-06 | 2.38E-03 |
| transcription regulator activity | 1989 | 93 | 59.91 | 1.55 | + | 3.62E-05 | 5.96E-03 |
| sequence-specific DNA binding | 1709 | 79 | 51.47 | 1.53 | + | 2.28E-04 | 3.03E-02 |
| metal ion binding | 4289 | 187 | 129.18 | 1.45 | + | 8.27E-08 | 4.39E-05 |
| cation binding | 4376 | 190 | 131.80 | 1.44 | + | 7.87E-08 | 4.70E-05 |
| protein binding | 14393 | 494 | 433.49 | 1.14 | + | 1.23E-07 | 5.35E-05 |
| Unclassified | 2468 | 22 | 74.33 | .30 | - | 2.64E-13 | 3.15E-10 |
| G protein-coupled receptor activity | 884 | 5 | 26.62 | .19 | - | 6.71E-07 | 2.47E-04 |
| transmembrane signaling receptor activity | 1331 | 16 | 40.09 | .40 | - | 1.80E-05 | 3.91E-03 |
| signaling receptor activity | 1518 | 19 | 45.72 | .42 | - | 9.17E-06 | 2.31E-03 |
| molecular transducer activity | 1518 | 19 | 45.72 | .42 | - | 9.17E-06 | 2.43E-03 |
| olfactory receptor activity | 440 | 0 | 13.25 | < 0.01 | - | 4.30E-06 | 1.28E-03 |

S12. CELLULAR COMPONENTS

|  | Homo sapiens | RESULTS | | | | | |
| --- | --- | --- | --- | --- | --- | --- | --- |
| GO cellular component complete | # | # | expected | Fold Enrichment | +/- | raw P value | FDR |
| intraciliary transport particle B | 18 | 6 | .54 | 11.07 | + | 5.23E-05 | 4.20E-03 |
| intraciliary transport particle | 26 | 8 | .78 | 10.22 | + | 4.73E-06 | 5.28E-04 |
| complex of collagen trimers | 21 | 5 | .63 | 7.91 | + | 8.31E-04 | 4.90E-02 |
| cellular anatomical entity | 18825 | 605 | 566.98 | 1.07 | + | 1.72E-08 | 2.46E-06 |
| ciliary tip | 47 | 9 | 1.42 | 6.36 | + | 3.27E-05 | 2.86E-03 |
| cilium | 683 | 38 | 20.57 | 1.85 | + | 4.47E-04 | 2.99E-02 |
| organelle | 13908 | 522 | 418.89 | 1.25 | + | 1.13E-19 | 5.69E-17 |
| ciliary transition zone | 73 | 10 | 2.20 | 4.55 | + | 1.52E-04 | 1.09E-02 |
| ciliary basal body | 157 | 17 | 4.73 | 3.60 | + | 1.47E-05 | 1.47E-03 |
| microtubule organizing center | 799 | 51 | 24.06 | 2.12 | + | 1.21E-06 | 1.43E-04 |
| microtubule cytoskeleton | 1285 | 65 | 38.70 | 1.68 | + | 7.73E-05 | 5.97E-03 |
| intracellular non-membrane-bounded organelle | 5304 | 199 | 159.75 | 1.25 | + | 5.84E-04 | 3.78E-02 |
| intracellular organelle | 13102 | 508 | 394.61 | 1.29 | + | 4.38E-22 | 4.39E-19 |
| intracellular anatomical structure | 14899 | 564 | 448.73 | 1.26 | + | 2.78E-28 | 5.58E-25 |
| non-membrane-bounded organelle | 5307 | 199 | 159.84 | 1.25 | + | 5.86E-04 | 3.67E-02 |
| nuclear speck | 403 | 26 | 12.14 | 2.14 | + | 6.68E-04 | 4.06E-02 |
| nuclear body | 796 | 51 | 23.97 | 2.13 | + | 1.12E-06 | 1.40E-04 |
| nucleoplasm | 3991 | 192 | 120.20 | 1.60 | + | 1.47E-11 | 4.22E-09 |
| nuclear lumen | 4992 | 223 | 150.35 | 1.48 | + | 1.88E-10 | 4.71E-08 |
| intracellular organelle lumen | 6118 | 260 | 184.26 | 1.41 | + | 2.50E-10 | 5.02E-08 |
| organelle lumen | 6118 | 260 | 184.26 | 1.41 | + | 2.50E-10 | 5.57E-08 |
| membrane-enclosed lumen | 6118 | 260 | 184.26 | 1.41 | + | 2.50E-10 | 4.56E-08 |
| nucleus | 7603 | 331 | 228.99 | 1.45 | + | 4.40E-16 | 1.47E-13 |
| intracellular membrane-bounded organelle | 11304 | 456 | 340.46 | 1.34 | + | 1.45E-20 | 9.72E-18 |
| membrane-bounded organelle | 12791 | 488 | 385.25 | 1.27 | + | 8.98E-18 | 3.60E-15 |
| centrosome | 602 | 38 | 18.13 | 2.10 | + | 3.84E-05 | 3.21E-03 |
| cytosol | 5305 | 211 | 159.78 | 1.32 | + | 8.05E-06 | 8.50E-04 |
| cytoplasm | 11958 | 426 | 360.16 | 1.18 | + | 1.15E-07 | 1.54E-05 |
| integral component of membrane | 5792 | 128 | 174.45 | .73 | - | 2.94E-05 | 2.68E-03 |
| intrinsic component of membrane | 5948 | 131 | 179.14 | .73 | - | 1.86E-05 | 1.77E-03 |
| intrinsic component of plasma membrane | 1734 | 30 | 52.23 | .57 | - | 8.66E-04 | 4.96E-02 |
| plasma membrane | 5837 | 134 | 175.80 | .76 | - | 2.04E-04 | 1.41E-02 |
| cell periphery | 5954 | 136 | 179.33 | .76 | - | 1.30E-04 | 9.64E-03 |
| Unclassified | 1887 | 19 | 56.83 | .33 | - | 5.22E-09 | 8.72E-07 |

*B2. Lower SE in parous.*

S13. BIOLOGICAL PROCESSES

|  | Homo sapiens | RESULTS | | | | | |
| --- | --- | --- | --- | --- | --- | --- | --- |
| GO biological process complete | # | # | expected | Fold Enrichment | +/- | raw P value | FDR |
| positive regulation of fibroblast apoptotic process | 8 | 5 | .32 | 15.55 | + | 8.45E-05 | 1.28E-02 |
| regulation of biological process | 11960 | 532 | 480.67 | 1.11 | + | 4.14E-04 | 4.69E-02 |
| biological regulation | 12645 | 568 | 508.20 | 1.12 | + | 3.20E-05 | 5.65E-03 |
| positive regulation of cellular process | 5695 | 304 | 228.88 | 1.33 | + | 3.17E-08 | 1.86E-05 |
| positive regulation of biological process | 6290 | 322 | 252.79 | 1.27 | + | 6.15E-07 | 2.17E-04 |
| purine nucleobase biosynthetic process | 10 | 5 | .40 | 12.44 | + | 1.85E-04 | 2.46E-02 |
| organic cyclic compound metabolic process | 3220 | 187 | 129.41 | 1.45 | + | 3.30E-07 | 1.25E-04 |
| organic substance metabolic process | 8011 | 452 | 321.96 | 1.40 | + | 6.77E-19 | 3.58E-15 |
| metabolic process | 8592 | 478 | 345.31 | 1.38 | + | 1.74E-19 | 2.75E-15 |
| organic substance biosynthetic process | 2799 | 168 | 112.49 | 1.49 | + | 1.98E-07 | 8.07E-05 |
| biosynthetic process | 2861 | 171 | 114.98 | 1.49 | + | 1.95E-07 | 8.12E-05 |
| heterocycle metabolic process | 2937 | 175 | 118.04 | 1.48 | + | 1.55E-07 | 6.83E-05 |
| cellular metabolic process | 7784 | 441 | 312.84 | 1.41 | + | 1.65E-18 | 6.54E-15 |
| cellular process | 15598 | 733 | 626.88 | 1.17 | + | 5.54E-19 | 4.39E-15 |
| cellular biosynthetic process | 2703 | 167 | 108.63 | 1.54 | + | 4.00E-08 | 2.12E-05 |
| cellular nitrogen compound biosynthetic process | 1578 | 99 | 63.42 | 1.56 | + | 2.20E-05 | 4.02E-03 |
| cellular nitrogen compound metabolic process | 3405 | 213 | 136.85 | 1.56 | + | 5.39E-11 | 6.58E-08 |
| nitrogen compound metabolic process | 7091 | 409 | 284.99 | 1.44 | + | 6.76E-18 | 2.15E-14 |
| organonitrogen compound metabolic process | 5444 | 303 | 218.79 | 1.38 | + | 3.99E-10 | 3.96E-07 |
| cellular aromatic compound metabolic process | 2987 | 180 | 120.05 | 1.50 | + | 4.66E-08 | 2.24E-05 |
| organonitrogen compound biosynthetic process | 1403 | 92 | 56.39 | 1.63 | + | 1.02E-05 | 2.06E-03 |
| nucleobase-containing compound metabolic process | 2743 | 167 | 110.24 | 1.51 | + | 8.83E-08 | 4.00E-05 |
| primary metabolic process | 7578 | 428 | 304.56 | 1.41 | + | 2.03E-17 | 5.38E-14 |
| polyamine metabolic process | 19 | 7 | .76 | 9.17 | + | 4.33E-05 | 7.39E-03 |
| microtubule anchoring | 26 | 7 | 1.04 | 6.70 | + | 2.22E-04 | 2.89E-02 |
| organelle organization | 3586 | 209 | 144.12 | 1.45 | + | 3.04E-08 | 1.85E-05 |
| cellular component organization | 5703 | 313 | 229.20 | 1.37 | + | 7.19E-10 | 6.71E-07 |
| cellular component organization or biogenesis | 5922 | 331 | 238.00 | 1.39 | + | 1.40E-11 | 2.02E-08 |
| antigen processing and presentation of exogenous peptide antigen | 178 | 19 | 7.15 | 2.66 | + | 2.40E-04 | 3.07E-02 |
| antigen processing and presentation of exogenous antigen | 185 | 19 | 7.44 | 2.56 | + | 3.73E-04 | 4.29E-02 |
| response to unfolded protein | 169 | 18 | 6.79 | 2.65 | + | 3.54E-04 | 4.16E-02 |
| response to topologically incorrect protein | 190 | 20 | 7.64 | 2.62 | + | 1.97E-04 | 2.60E-02 |
| response to stress | 3663 | 189 | 147.22 | 1.28 | + | 3.17E-04 | 3.87E-02 |
| cytoskeleton-dependent intracellular transport | 193 | 20 | 7.76 | 2.58 | + | 2.38E-04 | 3.07E-02 |
| intracellular transport | 1526 | 108 | 61.33 | 1.76 | + | 3.33E-08 | 1.82E-05 |
| establishment of localization in cell | 2378 | 160 | 95.57 | 1.67 | + | 2.96E-10 | 3.36E-07 |
| cellular localization | 3005 | 195 | 120.77 | 1.61 | + | 2.79E-11 | 3.69E-08 |
| localization | 5873 | 313 | 236.04 | 1.33 | + | 1.75E-08 | 1.11E-05 |
| establishment of localization | 4713 | 262 | 189.42 | 1.38 | + | 1.73E-08 | 1.15E-05 |
| transport | 4574 | 254 | 183.83 | 1.38 | + | 3.32E-08 | 1.88E-05 |
| response to endoplasmic reticulum stress | 262 | 26 | 10.53 | 2.47 | + | 6.86E-05 | 1.09E-02 |
| cellular response to stress | 1761 | 116 | 70.77 | 1.64 | + | 3.34E-07 | 1.23E-04 |
| regulation of cell cycle G2/M phase transition | 215 | 21 | 8.64 | 2.43 | + | 4.37E-04 | 4.92E-02 |
| regulation of cell cycle process | 769 | 58 | 30.91 | 1.88 | + | 1.23E-05 | 2.41E-03 |
| regulation of cell cycle | 1209 | 84 | 48.59 | 1.73 | + | 3.25E-06 | 8.05E-04 |
| rRNA metabolic process | 270 | 25 | 10.85 | 2.30 | + | 3.22E-04 | 3.91E-02 |
| RNA metabolic process | 1619 | 106 | 65.07 | 1.63 | + | 1.72E-06 | 4.86E-04 |
| nucleic acid metabolic process | 2241 | 138 | 90.07 | 1.53 | + | 8.48E-07 | 2.86E-04 |
| macromolecule metabolic process | 6333 | 367 | 254.52 | 1.44 | + | 1.42E-15 | 3.21E-12 |
| urogenital system development | 331 | 28 | 13.30 | 2.10 | + | 4.45E-04 | 4.93E-02 |
| neutrophil degranulation | 482 | 40 | 19.37 | 2.06 | + | 4.29E-05 | 7.40E-03 |
| neutrophil mediated immunity | 493 | 40 | 19.81 | 2.02 | + | 8.36E-05 | 1.28E-02 |
| myeloid leukocyte mediated immunity | 514 | 41 | 20.66 | 1.98 | + | 7.62E-05 | 1.19E-02 |
| leukocyte mediated immunity | 760 | 52 | 30.54 | 1.70 | + | 3.80E-04 | 4.33E-02 |
| neutrophil activation involved in immune response | 486 | 40 | 19.53 | 2.05 | + | 4.79E-05 | 8.00E-03 |
| neutrophil activation | 494 | 40 | 19.85 | 2.01 | + | 8.49E-05 | 1.27E-02 |
| granulocyte activation | 498 | 40 | 20.01 | 2.00 | + | 9.11E-05 | 1.34E-02 |
| myeloid leukocyte activation | 584 | 44 | 23.47 | 1.87 | + | 1.38E-04 | 1.90E-02 |
| leukocyte activation | 931 | 68 | 37.42 | 1.82 | + | 6.69E-06 | 1.52E-03 |
| cell activation | 1076 | 72 | 43.24 | 1.66 | + | 5.30E-05 | 8.66E-03 |
| myeloid cell activation involved in immune response | 519 | 41 | 20.86 | 1.97 | + | 8.73E-05 | 1.29E-02 |
| leukocyte activation involved in immune response | 622 | 46 | 25.00 | 1.84 | + | 1.45E-04 | 1.99E-02 |
| cell activation involved in immune response | 626 | 47 | 25.16 | 1.87 | + | 9.52E-05 | 1.39E-02 |
| leukocyte degranulation | 504 | 42 | 20.26 | 2.07 | + | 2.38E-05 | 4.25E-03 |
| regulated exocytosis | 691 | 52 | 27.77 | 1.87 | + | 3.87E-05 | 6.75E-03 |
| exocytosis | 788 | 54 | 31.67 | 1.71 | + | 3.33E-04 | 3.98E-02 |
| vesicle-mediated transport | 1972 | 124 | 79.25 | 1.56 | + | 1.60E-06 | 4.79E-04 |
| regulation of mitotic cell cycle | 597 | 49 | 23.99 | 2.04 | + | 9.61E-06 | 1.96E-03 |
| ribonucleoprotein complex biogenesis | 468 | 38 | 18.81 | 2.02 | + | 9.59E-05 | 1.38E-02 |
| cellular component biogenesis | 2654 | 158 | 106.66 | 1.48 | + | 9.57E-07 | 3.10E-04 |
| viral process | 845 | 63 | 33.96 | 1.86 | + | 8.57E-06 | 1.81E-03 |
| symbiotic process | 936 | 66 | 37.62 | 1.75 | + | 2.33E-05 | 4.20E-03 |
| proteolysis involved in cellular protein catabolic process | 604 | 44 | 24.27 | 1.81 | + | 3.73E-04 | 4.32E-02 |
| cellular protein catabolic process | 637 | 47 | 25.60 | 1.84 | + | 1.61E-04 | 2.18E-02 |
| cellular protein metabolic process | 3822 | 226 | 153.61 | 1.47 | + | 1.98E-09 | 1.66E-06 |
| cellular macromolecule metabolic process | 5145 | 306 | 206.78 | 1.48 | + | 1.01E-13 | 2.01E-10 |
| protein metabolic process | 4407 | 246 | 177.12 | 1.39 | + | 4.47E-08 | 2.22E-05 |
| cellular macromolecule catabolic process | 926 | 70 | 37.22 | 1.88 | + | 1.26E-06 | 4.00E-04 |
| macromolecule catabolic process | 1060 | 77 | 42.60 | 1.81 | + | 1.66E-06 | 4.79E-04 |
| organic substance catabolic process | 1782 | 112 | 71.62 | 1.56 | + | 4.78E-06 | 1.13E-03 |
| catabolic process | 2103 | 129 | 84.52 | 1.53 | + | 3.12E-06 | 7.97E-04 |
| cellular catabolic process | 1830 | 116 | 73.55 | 1.58 | + | 2.44E-06 | 6.45E-04 |
| protein catabolic process | 691 | 50 | 27.77 | 1.80 | + | 1.35E-04 | 1.88E-02 |
| protein localization to organelle | 779 | 55 | 31.31 | 1.76 | + | 1.08E-04 | 1.53E-02 |
| cellular protein localization | 1643 | 97 | 66.03 | 1.47 | + | 2.65E-04 | 3.31E-02 |
| cellular macromolecule localization | 1652 | 97 | 66.39 | 1.46 | + | 2.79E-04 | 3.46E-02 |
| macromolecule localization | 2561 | 143 | 102.93 | 1.39 | + | 7.66E-05 | 1.18E-02 |
| protein localization | 2198 | 130 | 88.34 | 1.47 | + | 1.42E-05 | 2.75E-03 |
| RNA processing | 929 | 64 | 37.34 | 1.71 | + | 6.49E-05 | 1.04E-02 |
| gene expression | 2105 | 146 | 84.60 | 1.73 | + | 3.03E-10 | 3.20E-07 |
| intracellular protein transport | 992 | 65 | 39.87 | 1.63 | + | 2.13E-04 | 2.80E-02 |
| protein transport | 1509 | 102 | 60.65 | 1.68 | + | 7.66E-07 | 2.64E-04 |
| establishment of protein localization | 1593 | 108 | 64.02 | 1.69 | + | 2.62E-07 | 1.04E-04 |
| peptide transport | 1540 | 106 | 61.89 | 1.71 | + | 1.70E-07 | 7.28E-05 |
| amide transport | 1575 | 107 | 63.30 | 1.69 | + | 3.16E-07 | 1.22E-04 |
| nitrogen compound transport | 1861 | 125 | 74.79 | 1.67 | + | 4.28E-08 | 2.19E-05 |
| organic substance transport | 2229 | 135 | 89.58 | 1.51 | + | 3.18E-06 | 8.02E-04 |
| cellular macromolecule biosynthetic process | 1636 | 105 | 65.75 | 1.60 | + | 4.93E-06 | 1.15E-03 |
| macromolecule biosynthetic process | 1682 | 108 | 67.60 | 1.60 | + | 3.50E-06 | 8.53E-04 |
| positive regulation of cellular component organization | 1161 | 72 | 46.66 | 1.54 | + | 4.44E-04 | 4.96E-02 |
| protein-containing complex subunit organization | 1547 | 94 | 62.17 | 1.51 | + | 1.07E-04 | 1.52E-02 |
| positive regulation of signal transduction | 1564 | 95 | 62.86 | 1.51 | + | 1.12E-04 | 1.57E-02 |
| regulation of signaling | 3479 | 194 | 139.82 | 1.39 | + | 2.47E-06 | 6.43E-04 |
| regulation of cell communication | 3443 | 190 | 138.37 | 1.37 | + | 6.93E-06 | 1.53E-03 |
| regulation of signal transduction | 3078 | 174 | 123.70 | 1.41 | + | 5.21E-06 | 1.20E-03 |
| regulation of response to stimulus | 4287 | 217 | 172.29 | 1.26 | + | 2.60E-04 | 3.31E-02 |
| regulation of intracellular signal transduction | 1787 | 105 | 71.82 | 1.46 | + | 1.69E-04 | 2.27E-02 |
| regulation of developmental process | 2469 | 144 | 99.23 | 1.45 | + | 8.53E-06 | 1.83E-03 |
| positive regulation of macromolecule biosynthetic process | 1861 | 107 | 74.79 | 1.43 | + | 2.84E-04 | 3.49E-02 |
| regulation of macromolecule metabolic process | 6491 | 328 | 260.87 | 1.26 | + | 1.65E-06 | 4.85E-04 |
| regulation of metabolic process | 7025 | 350 | 282.33 | 1.24 | + | 1.86E-06 | 5.18E-04 |
| positive regulation of biosynthetic process | 1998 | 113 | 80.30 | 1.41 | + | 3.48E-04 | 4.12E-02 |
| positive regulation of metabolic process | 3861 | 212 | 155.17 | 1.37 | + | 2.03E-06 | 5.46E-04 |
| positive regulation of macromolecule metabolic process | 3571 | 195 | 143.52 | 1.36 | + | 9.38E-06 | 1.96E-03 |
| cellular protein modification process | 3188 | 182 | 128.13 | 1.42 | + | 1.37E-06 | 4.25E-04 |
| protein modification process | 3188 | 182 | 128.13 | 1.42 | + | 1.37E-06 | 4.17E-04 |
| macromolecule modification | 3412 | 195 | 137.13 | 1.42 | + | 4.79E-07 | 1.73E-04 |
| cellular component assembly | 2394 | 136 | 96.21 | 1.41 | + | 5.86E-05 | 9.49E-03 |
| regulation of cellular protein metabolic process | 2668 | 149 | 107.23 | 1.39 | + | 5.23E-05 | 8.64E-03 |
| regulation of cellular metabolic process | 6304 | 313 | 253.36 | 1.24 | + | 1.76E-05 | 3.33E-03 |
| regulation of protein metabolic process | 2806 | 156 | 112.77 | 1.38 | + | 4.79E-05 | 8.08E-03 |
| regulation of primary metabolic process | 6086 | 305 | 244.60 | 1.25 | + | 1.03E-05 | 2.04E-03 |
| regulation of nitrogen compound metabolic process | 5895 | 296 | 236.92 | 1.25 | + | 1.44E-05 | 2.75E-03 |
| positive regulation of nitrogen compound metabolic process | 3206 | 179 | 128.85 | 1.39 | + | 7.44E-06 | 1.62E-03 |
| positive regulation of cellular metabolic process | 3392 | 187 | 136.32 | 1.37 | + | 9.52E-06 | 1.96E-03 |
| negative regulation of macromolecule metabolic process | 2908 | 156 | 116.87 | 1.33 | + | 2.61E-04 | 3.29E-02 |
| negative regulation of biological process | 5592 | 289 | 224.74 | 1.29 | + | 1.87E-06 | 5.11E-04 |
| regulation of biological quality | 4033 | 210 | 162.09 | 1.30 | + | 7.47E-05 | 1.17E-02 |
| negative regulation of cellular process | 4952 | 254 | 199.02 | 1.28 | + | 2.08E-05 | 3.84E-03 |
| G protein-coupled receptor signaling pathway | 1323 | 25 | 53.17 | .47 | - | 2.07E-05 | 3.86E-03 |
| Unclassified | 2839 | 51 | 114.10 | .45 | - | 7.50E-12 | 1.32E-08 |
| detection of chemical stimulus involved in sensory perception of smell | 440 | 0 | 17.68 | < 0.01 | - | 5.10E-08 | 2.38E-05 |
| detection of chemical stimulus involved in sensory perception | 485 | 0 | 19.49 | < 0.01 | - | 6.71E-09 | 5.07E-06 |
| detection of stimulus involved in sensory perception | 550 | 1 | 22.10 | .05 | - | 1.49E-08 | 1.07E-05 |
| sensory perception | 981 | 14 | 39.43 | .36 | - | 4.60E-06 | 1.11E-03 |
| nervous system process | 1403 | 26 | 56.39 | .46 | - | 6.86E-06 | 1.53E-03 |
| system process | 2066 | 53 | 83.03 | .64 | - | 3.60E-04 | 4.20E-02 |
| detection of stimulus | 712 | 6 | 28.62 | .21 | - | 9.02E-07 | 2.98E-04 |
| detection of chemical stimulus | 521 | 0 | 20.94 | < 0.01 | - | 2.18E-09 | 1.73E-06 |
| sensory perception of chemical stimulus | 538 | 0 | 21.62 | < 0.01 | - | 9.38E-10 | 8.27E-07 |
| sensory perception of smell | 466 | 0 | 18.73 | < 0.01 | - | 1.49E-08 | 1.03E-05 |
| keratinization | 226 | 0 | 9.08 | < 0.01 | - | 3.24E-04 | 3.89E-02 |

S14. MOLECULAR FUNCTIONS

|  | Homo sapiens | RESULTS | | | | | |
| --- | --- | --- | --- | --- | --- | --- | --- |
| GO molecular function complete | # | # | expected | Fold Enrichment | +/- | raw P value | FDR |
| transcription corepressor activity | 189 | 22 | 7.60 | 2.90 | + | 2.45E-05 | 6.90E-03 |
| transcription coregulator activity | 489 | 44 | 19.65 | 2.24 | + | 3.22E-06 | 1.10E-03 |
| chromatin binding | 582 | 48 | 23.39 | 2.05 | + | 7.86E-06 | 2.35E-03 |
| binding | 16634 | 774 | 668.52 | 1.16 | + | 5.49E-23 | 1.31E-19 |
| RNA binding | 1691 | 128 | 67.96 | 1.88 | + | 2.19E-11 | 2.09E-08 |
| nucleic acid binding | 4007 | 220 | 161.04 | 1.37 | + | 1.16E-06 | 4.26E-04 |
| organic cyclic compound binding | 6066 | 322 | 243.79 | 1.32 | + | 1.50E-08 | 1.03E-05 |
| heterocyclic compound binding | 5977 | 320 | 240.22 | 1.33 | + | 6.55E-09 | 5.21E-06 |
| transferase activity, transferring phosphorus-containing groups | 937 | 63 | 37.66 | 1.67 | + | 1.41E-04 | 2.69E-02 |
| transferase activity | 2347 | 143 | 94.33 | 1.52 | + | 1.02E-06 | 4.07E-04 |
| catalytic activity | 5840 | 307 | 234.71 | 1.31 | + | 1.20E-07 | 5.76E-05 |
| protein-containing complex binding | 1272 | 83 | 51.12 | 1.62 | + | 3.00E-05 | 7.95E-03 |
| enzyme binding | 2302 | 137 | 92.52 | 1.48 | + | 5.78E-06 | 1.84E-03 |
| protein binding | 14393 | 709 | 578.45 | 1.23 | + | 2.30E-24 | 1.10E-20 |
| purine ribonucleotide binding | 1929 | 112 | 77.53 | 1.44 | + | 1.37E-04 | 2.72E-02 |
| purine nucleotide binding | 1943 | 113 | 78.09 | 1.45 | + | 1.14E-04 | 2.36E-02 |
| nucleotide binding | 2184 | 125 | 87.77 | 1.42 | + | 9.72E-05 | 2.21E-02 |
| nucleoside phosphate binding | 2185 | 125 | 87.81 | 1.42 | + | 9.77E-05 | 2.12E-02 |
| ribonucleotide binding | 1946 | 112 | 78.21 | 1.43 | + | 1.86E-04 | 3.42E-02 |
| purine ribonucleoside triphosphate binding | 1859 | 107 | 74.71 | 1.43 | + | 2.81E-04 | 4.79E-02 |
| Unclassified | 2468 | 25 | 99.19 | .25 | - | 8.93E-20 | 1.42E-16 |
| G protein-coupled receptor activity | 884 | 8 | 35.53 | .23 | - | 6.24E-08 | 3.31E-05 |
| transmembrane signaling receptor activity | 1331 | 20 | 53.49 | .37 | - | 1.82E-07 | 7.90E-05 |
| signaling receptor activity | 1518 | 32 | 61.01 | .52 | - | 5.03E-05 | 1.20E-02 |
| molecular transducer activity | 1518 | 32 | 61.01 | .52 | - | 5.03E-05 | 1.26E-02 |
| gated channel activity | 344 | 2 | 13.83 | .14 | - | 2.08E-04 | 3.68E-02 |
| olfactory receptor activity | 440 | 0 | 17.68 | < 0.01 | - | 5.10E-08 | 3.05E-05 |

S15. CELLULAR COMPONENTS

|  | Homo sapiens | RESULTS | | | | | |  |
| --- | --- | --- | --- | --- | --- | --- | --- | --- |
| GO cellular component complete | # | # | expected | Fold Enrichment | +/- | raw P value | FDR | |
| MHC class I protein complex | 9 | 4 | .36 | 11.06 | + | 1.20E-03 | 3.64E-02 | |
| MHC protein complex | 28 | 6 | 1.13 | 5.33 | + | 1.74E-03 | 4.99E-02 | |
| membrane | 9948 | 453 | 399.81 | 1.13 | + | 3.19E-04 | 1.28E-02 | |
| cellular anatomical entity | 18825 | 813 | 756.58 | 1.07 | + | 1.50E-13 | 2.74E-11 | |
| protein-containing complex | 5563 | 291 | 223.58 | 1.30 | + | 5.35E-07 | 4.29E-05 | |
| integral component of lumenal side of endoplasmic reticulum membrane | 29 | 7 | 1.17 | 6.01 | + | 3.92E-04 | 1.46E-02 | |
| intrinsic component of organelle membrane | 402 | 31 | 16.16 | 1.92 | + | 9.51E-04 | 3.03E-02 | |
| organelle membrane | 3642 | 228 | 146.37 | 1.56 | + | 7.26E-12 | 9.71E-10 | |
| membrane-bounded organelle | 12791 | 668 | 514.07 | 1.30 | + | 3.82E-29 | 2.56E-26 | |
| organelle | 13908 | 703 | 558.96 | 1.26 | + | 3.99E-28 | 2.00E-25 | |
| endoplasmic reticulum membrane | 1142 | 84 | 45.90 | 1.83 | + | 3.56E-07 | 3.10E-05 | |
| endoplasmic reticulum subcompartment | 1147 | 84 | 46.10 | 1.82 | + | 3.81E-07 | 3.19E-05 | |
| organelle subcompartment | 1798 | 123 | 72.26 | 1.70 | + | 1.88E-08 | 2.10E-06 | |
| intracellular organelle | 13102 | 664 | 526.57 | 1.26 | + | 9.24E-24 | 3.09E-21 | |
| intracellular anatomical structure | 14899 | 744 | 598.79 | 1.24 | + | 1.22E-32 | 2.45E-29 | |
| endoplasmic reticulum | 2009 | 126 | 80.74 | 1.56 | + | 1.45E-06 | 1.04E-04 | |
| cytoplasm | 11958 | 640 | 480.59 | 1.33 | + | 1.75E-29 | 1.75E-26 | |
| intracellular membrane-bounded organelle | 11304 | 600 | 454.31 | 1.32 | + | 4.98E-24 | 2.00E-21 | |
| endomembrane system | 4664 | 257 | 187.45 | 1.37 | + | 5.23E-08 | 5.52E-06 | |
| nuclear outer membrane-endoplasmic reticulum membrane network | 1165 | 84 | 46.82 | 1.79 | + | 6.93E-07 | 5.35E-05 | |
| integral component of organelle membrane | 372 | 31 | 14.95 | 2.07 | + | 3.30E-04 | 1.27E-02 | |
| lumenal side of endoplasmic reticulum membrane | 29 | 7 | 1.17 | 6.01 | + | 3.92E-04 | 1.43E-02 | |
| lumenal side of membrane | 37 | 8 | 1.49 | 5.38 | + | 2.91E-04 | 1.22E-02 | |
| azurophil granule membrane | 58 | 10 | 2.33 | 4.29 | + | 2.68E-04 | 1.17E-02 | |
| secretory granule membrane | 306 | 27 | 12.30 | 2.20 | + | 2.78E-04 | 1.18E-02 | |
| cytoplasmic vesicle | 2453 | 137 | 98.59 | 1.39 | + | 1.11E-04 | 5.45E-03 | |
| intracellular vesicle | 2458 | 138 | 98.79 | 1.40 | + | 8.89E-05 | 4.46E-03 | |
| vesicle | 3939 | 223 | 158.31 | 1.41 | + | 9.10E-08 | 8.69E-06 | |
| bounding membrane of organelle | 2172 | 137 | 87.29 | 1.57 | + | 3.14E-07 | 2.86E-05 | |
| lysosomal membrane | 385 | 31 | 15.47 | 2.00 | + | 4.65E-04 | 1.64E-02 | |
| lytic vacuole membrane | 385 | 31 | 15.47 | 2.00 | + | 4.65E-04 | 1.66E-02 | |
| vacuolar membrane | 439 | 33 | 17.64 | 1.87 | + | 1.01E-03 | 3.16E-02 | |
| azurophil granule | 154 | 16 | 6.19 | 2.59 | + | 9.40E-04 | 3.09E-02 | |
| primary lysosome | 154 | 16 | 6.19 | 2.59 | + | 9.40E-04 | 3.04E-02 | |
| cis-Golgi network | 71 | 11 | 2.85 | 3.85 | + | 3.07E-04 | 1.26E-02 | |
| Golgi apparatus | 1619 | 107 | 65.07 | 1.64 | + | 9.12E-07 | 6.78E-05 | |
| ER to Golgi transport vesicle membrane | 61 | 9 | 2.45 | 3.67 | + | 1.45E-03 | 4.21E-02 | |
| tertiary granule membrane | 73 | 10 | 2.93 | 3.41 | + | 1.33E-03 | 4.00E-02 | |
| centriolar satellite | 97 | 13 | 3.90 | 3.33 | + | 3.26E-04 | 1.28E-02 | |
| centrosome | 602 | 45 | 24.19 | 1.86 | + | 1.61E-04 | 7.34E-03 | |
| microtubule organizing center | 799 | 53 | 32.11 | 1.65 | + | 7.22E-04 | 2.50E-02 | |
| microtubule cytoskeleton | 1285 | 83 | 51.64 | 1.61 | + | 4.44E-05 | 2.34E-03 | |
| cytoskeleton | 2311 | 133 | 92.88 | 1.43 | + | 4.46E-05 | 2.29E-03 | |
| intracellular non-membrane-bounded organelle | 5304 | 273 | 213.17 | 1.28 | + | 6.07E-06 | 3.69E-04 | |
| non-membrane-bounded organelle | 5307 | 273 | 213.29 | 1.28 | + | 6.13E-06 | 3.62E-04 | |
| Golgi membrane | 777 | 59 | 31.23 | 1.89 | + | 8.77E-06 | 5.03E-04 | |
| Golgi apparatus subcompartment | 891 | 67 | 35.81 | 1.87 | + | 2.82E-06 | 1.77E-04 | |
| ribonucleoprotein complex | 700 | 53 | 28.13 | 1.88 | + | 2.82E-05 | 1.53E-03 | |
| cell leading edge | 429 | 32 | 17.24 | 1.86 | + | 1.36E-03 | 4.01E-02 | |
| focal adhesion | 417 | 31 | 16.76 | 1.85 | + | 1.76E-03 | 4.97E-02 | |
| nucleolus | 945 | 67 | 37.98 | 1.76 | + | 1.73E-05 | 9.64E-04 | |
| nuclear lumen | 4992 | 282 | 200.63 | 1.41 | + | 6.10E-10 | 7.19E-08 | |
| intracellular organelle lumen | 6118 | 343 | 245.88 | 1.39 | + | 3.18E-12 | 4.90E-10 | |
| organelle lumen | 6118 | 343 | 245.88 | 1.39 | + | 3.18E-12 | 5.31E-10 | |
| membrane-enclosed lumen | 6118 | 343 | 245.88 | 1.39 | + | 3.18E-12 | 4.55E-10 | |
| nucleus | 7603 | 395 | 305.56 | 1.29 | + | 6.02E-10 | 7.55E-08 | |
| transferase complex | 795 | 54 | 31.95 | 1.69 | + | 3.56E-04 | 1.35E-02 | |
| catalytic complex | 1411 | 101 | 56.71 | 1.78 | + | 5.39E-08 | 5.40E-06 | |
| mitochondrial envelope | 787 | 52 | 31.63 | 1.64 | + | 9.19E-04 | 3.07E-02 | |
| organelle envelope | 1238 | 78 | 49.76 | 1.57 | + | 1.57E-04 | 7.32E-03 | |
| envelope | 1238 | 78 | 49.76 | 1.57 | + | 1.57E-04 | 7.49E-03 | |
| nucleoplasm | 3991 | 255 | 160.40 | 1.59 | + | 1.99E-14 | 4.98E-12 | |
| extracellular exosome | 2098 | 130 | 84.32 | 1.54 | + | 1.71E-06 | 1.11E-04 | |
| extracellular vesicle | 2119 | 131 | 85.16 | 1.54 | + | 1.46E-06 | 1.01E-04 | |
| extracellular organelle | 2121 | 131 | 85.24 | 1.54 | + | 1.49E-06 | 9.98E-05 | |
| cytosol | 5305 | 322 | 213.21 | 1.51 | + | 7.73E-16 | 2.21E-13 | |
| Unclassified | 1887 | 21 | 75.84 | .28 | - | 5.87E-14 | 1.31E-11 | |
| cation channel complex | 228 | 0 | 9.16 | < 0.01 | - | 1.99E-04 | 8.87E-03 | |
| ion channel complex | 308 | 2 | 12.38 | .16 | - | 8.76E-04 | 2.98E-02 | |
| transporter complex | 342 | 3 | 13.74 | .22 | - | 1.08E-03 | 3.34E-02 | |
